# Garbage in, garbage out: how reliable training data improved a virtual screening approach against SARS-CoV-2 MPro

**DOI:** 10.3389/fphar.2023.1193282

**Published:** 2023-06-22

**Authors:** Santiago M. Ruatta, Denis N. Prada Gori, Martín Fló Díaz, Franca Lorenzelli, Karen Perelmuter, Lucas N. Alberca, Carolina L. Bellera, Andrea Medeiros, Gloria V. López, Mariana Ingold, Williams Porcal, Estefanía Dibello, Irina Ihnatenko, Conrad Kunick, Marcelo Incerti, Martín Luzardo, Maximiliano Colobbio, Juan Carlos Ramos, Eduardo Manta, Lucía Minini, María Laura Lavaggi, Paola Hernández, Jonas Šarlauskas, César Sebastian Huerta García, Rafael Castillo, Alicia Hernández-Campos, Giovanni Ribaudo, Giuseppe Zagotto, Renzo Carlucci, Noelia S. Medrán, Guillermo R. Labadie, Maitena Martinez-Amezaga, Carina M. L. Delpiccolo, Ernesto G. Mata, Laura Scarone, Laura Posada, Gloria Serra, Theodora Calogeropoulou, Kyriakos Prousis, Anastasia Detsi, Mauricio Cabrera, Guzmán Alvarez, Adrián Aicardo, Verena Araújo, Cecilia Chavarría, Lucija Peterlin Mašič, Melisa E. Gantner, Manuel A. Llanos, Santiago Rodríguez, Luciana Gavernet, Soonju Park, Jinyeong Heo, Honggun Lee, Kyu-Ho Paul Park, Mariela Bollati-Fogolín, Otto Pritsch, David Shum, Alan Talevi, Marcelo A. Comini

**Affiliations:** ^1^ Laboratory Redox Biology of Trypanosomes, Institut Pasteur de Montevideo, Montevideo, Uruguay; ^2^ Facultad de Bioquímica y Ciencias Biológicas, Universidad Nacional del Litoral, Santa Fe, Argentina; ^3^ Laboratory of Bioactive Compound Research and Development (LIDeB), Faculty of Exact Sciences, National University of La Plata, Buenos Aires, Argentina; ^4^ Laboratory of Immunovirology, Institut Pasteur de Montevideo, Montevideo, Uruguay; ^5^ Departamento de Inmunobiología, Facultad de Medicina, Universidad de la República, Montevideo, Uruguay; ^6^ Cell Biology Unit, Institut Pasteur de Montevideo, Montevideo, Uruguay; ^7^ Consejo Nacional de Investigaciones Científicas y Técnicas (CONICET), Buenos Aires, Argentina; ^8^ Departamento de Bioquímica, Facultad de Medicina, Universidad de la República, Montevideo, Uruguay; ^9^ Departamento de Química Orgánica, Facultad de Química, Universidad de la República, Montevideo, Uruguay; ^10^ Vascular Biology and Drug Discovery Lab, Institut Pasteur de Montevideo, Montevideo, Uruguay; ^11^ PVZ—Center of Pharmaceutical Engineering, Institute of Medicinal and Pharmaceutical Chemistry, Technische Universität Braunschweig, Braunschweig, Germany; ^12^ Laboratorio de Química Fina, Facultad de Química, Instituto Polo Tecnológico de Pando, Universidad de la República, Montevideo, Uruguay; ^13^ Laboratorio de Química Biológica Ambiental, Sede Rivera, Centro Universitario Regional Noreste, Universidad de la República, Montevideo, Uruguay; ^14^ Departamento de Genética, Instituto de Investigaciones Biológicas Clemente Estable, Montevideo, Uruguay; ^15^ Life Sciences Centre, Department of Xenobiotic Biochemistry, Institute of Biochemistry, Vilnius University, Vilnius, Lithuania; ^16^ Departamento de Farmacia, Facultad de Química, Universidad Nacional Autónoma de México, Ciudad de Mexico, Mexico; ^17^ Department of Molecular and Translational Medicine, University of Brescia, Brescia, Italy; ^18^ Department of Pharmaceutical and Pharmacological Sciences, University of Padova, Padova, Italy; ^19^ Facultad de Ciencias Bioquímicas y Farmacéuticas, Instituto de Química Rosario (IQUIR) UNR, CONICET, Universidad Nacional de Rosario, Rosario, Argentina; ^20^ Institute of Chemical Biology, National Hellenic Research Foundation, Athens, Greece; ^21^ Laboratory of Organic Chemistry, School of Chemical Engineering, National Technical University of Athens, Athens, Greece; ^22^ Laboratorio de Moléculas Bioactivas, Departamento de Ciencias Biológicas, CENUR Litoral Norte, Universidad de la República, Paysandú, Uruguay; ^23^ Centro de Investigaciones Biomédicas (CEINBIO), Universidad de la República, Montevideo, Uruguay; ^24^ Departamento de Nutrición Clínica, Escuela de Nutrición, Universidad de la República, Montevideo, Uruguay; ^25^ Departamento de Alimentos, Escuela de Nutrición, Universidad de la República, Montevideo, Uruguay; ^26^ Fakulteta za farmacijo, Univerza v Ljubljani, Ljubljana, Slovenia; ^27^ Screening Discovery Platform, Institut Pasteur Korea, Seongnam, Republic of Korea

**Keywords:** *in silico* screening, coronavirus, COVID-19, protease, target-based, drug discovery, rubbish in rubbish out, artificial intelligence

## Abstract

**Introduction:** The identification of chemical compounds that interfere with SARS-CoV-2 replication continues to be a priority in several academic and pharmaceutical laboratories. Computational tools and approaches have the power to integrate, process and analyze multiple data in a short time. However, these initiatives may yield unrealistic results if the applied models are not inferred from reliable data and the resulting predictions are not confirmed by experimental evidence.

**Methods:** We undertook a drug discovery campaign against the essential major protease (MPro) from SARS-CoV-2, which relied on an *in silico* search strategy –performed in a large and diverse chemolibrary– complemented by experimental validation. The computational method comprises a recently reported ligand-based approach developed upon refinement/learning cycles, and structure-based approximations. Search models were applied to both retrospective (*in silico*) and prospective (experimentally confirmed) screening.

**Results:** The first generation of ligand-based models were fed by data, which to a great extent, had not been published in peer-reviewed articles. The first screening campaign performed with 188 compounds (46 *in silico* hits and 100 analogues, and 40 unrelated compounds: flavonols and pyrazoles) yielded three hits against MPro (IC_50_ ≤ 25 μM): two analogues of *in silico* hits (one glycoside and one benzo-thiazol) and one flavonol. A second generation of ligand-based models was developed based on this negative information and newly published peer-reviewed data for MPro inhibitors. This led to 43 new hit candidates belonging to different chemical families. From 45 compounds (28 in silico hits and 17 related analogues) tested in the second screening campaign, eight inhibited MPro with IC_50_ = 0.12–20 μM and five of them also impaired the proliferation of SARS-CoV-2 in Vero cells (EC_50_ 7–45 μM).

**Discussion:** Our study provides an example of a virtuous loop between computational and experimental approaches applied to target-focused drug discovery against a major and global pathogen, reaffirming the well-known “garbage in, garbage out” machine learning principle.

## 1 Introduction

Since few years ago, medicinal chemistry has been revolutionized by the application of artificial intelligence (AI) to research and development activities in the field of drug discovery, from target identification to rational drug design (Paul et al., 2021). Part of the power of AI and machine learning techniques relies on their capacity to perform multifactorial data processing and analyses that allow the identification of patterns hidden in large volumes of data. Such analyses can be applied to build predictive hypotheses that overcome the -usually frustrating and time-consuming- trial-and-error approaches. However, the success of intelligent algorithms in prediction-based approaches depends, to a great extent, on experimental information embracing the different hypothetical scenarios.

The scientific community reacted to the coronavirus disease 19 (COVID-19) pandemic by rapidly implementing different strategies to cope with the corresponding therapeutic and prophylactic needs. In this regard, AI methods have been applied for vaccine design and for the structure- and ligand-based prediction and identification of molecules (i.e., antibodies, peptides, small chemicals) targeting essential components of the causative agent of COVID-19 (reviewed by Bali and Bali, 2022; Floresta et al., 2022), the type 2 coronavirus that produces a severe acute respiratory syndrome (SARS-CoV-2). Several drugs with well-documented (e.g., Remdesivir) or so far unknown antiviral activity have been proposed by AI methods as repurposing candidates against SARS-CoV-2 (Bali and Bali, 2022).

Doubtlessly, vaccination and anti-viral chemotherapy contributed substantially to control virus dissemination and disease progression in a relatively short time since pandemic outbreak in late 2019 (Witek, 2021; Watson et al., 2022). However, the remarkable virus mutability along with the antibodies titer decay in the naturally or artificially immunized population accounts for the impossibility of eradicating SARS-CoV-2, which will persist as a global threat. Thus, drug discovery research against this (and other emerging pathogens) still deems important for feeding the pipeline of potential backup drug candidates.

Among the proteins encoded by the SARS-CoV-2 genome, the proteases (a chemotrypsin-like protease: 3CL-Pro or MPro, for Major Protease, and a Papain-Like Protease: PLPro) have attracted early attention as pharmacological targets because of their essential role in converting the long viral polypeptide into the single structural and non-structural proteins (Lv et al., 2022). MPro and PLPro are cysteine-proteases that are structurally unrelated and display sequence- and mechanistic-specificity for the hydrolysis of the peptide substrate. MPro cleaves the viral polypeptide at 11 sites. PLPro does it at three sites and also cleaves ubiquitin and Interferon–stimulated gene 15 (ISG-15), the latter playing an important modulatory role in host immune response and viral replication (Perng and Lenschow, 2018).

The pivotal role of MPro for SARS-CoV-2 replication has been confirmed by genetic and chemical approaches, and these laboratory-based findings translated into the recent approval of two clinical drugs targeting this protease: ensitrelvir (Mukae et al., 2022) and nirmatrelvir (Lamb, 2022).

With the aim to perfect AI methods applied to the discovery of small chemical compounds targeting MPro from SARS-CoV-2, here we report the results of a drug discovery campaign that combined ligand- and structure-based computer-aided strategies. The study was complemented by the experimental determination of the anti-MPro activity of the *in silico* candidates, and, for the confirmed hits, the evaluation of their anti-SARS-CoV-2 activity and cytotoxicity. The novelty of the findings is linked to the screening of an in-house chemical library. The iterative cycle “computer-wet lab-computer” proved key to perfecting the computational search methods and disclosed novel chemical scaffolds targeting the type-2 coronavirus major protease and replication.

## 2 Model development and validation

### 2.1 Dataset compilation and curation

#### 2.1.1 First ligand-based modelling

A dataset of compounds with reported IC_50_ values against MPro or reported residual enzyme activity at 10 or 20 µM was compiled from different sources. These included five original articles found in specialized literature (Jin et al., 2020; Ma et al., 2020; Su et al., 2020; Wenhao et al., 2020; Zhang et al., 2020) and data extracted from the publicly available COVID Moonshot database (Moonshot, 2021). The literature search and data compilation from the COVID Moonshot database was performed between July and October 2020. Compounds with IC_50_ < 10 µM were labelled as ACTIVE. In contrast, compounds with K_i_ or IC_50_ > 20 μM, percentage of inhibition <80% at 20 µM or percentage of enzyme inhibition <50% at 10 µM were labelled as INACTIVE. These complex criteria were used because at the time we initiated our study relatively few MPro inhibitors had been reported by different academic groups and the screening strategy used in each laboratory was quite variable: some of them reported inhibitors based on single point assays (e.g., at 10 or 20 µM) and others reported inhibitors based on dose-response studies. The dataset compounds, represented in SMILES format, were standardized using Standardizer 17.3.27.0 of JChem software (ChemAxon). Duplicate data and compounds with inconsistent labels from different sources were excluded. Finally, because only 0D-2D molecular descriptors were used for modelling purposes, when data associated with different optical isomers were reported only one of them was retained whenever both isomers belonged to the same activity class, and the compounds were disregarded if the isomers belonged to different activity classes. A total of 76 active compounds and 738 inactive compounds remained in the curated dataset.

#### 2.1.2 Second ligand-based modelling

A dataset of compounds with reported IC_50_ values against MPro or reported residual enzyme activity at 10, 20 or 50 µM was compiled from different sources. These included 18 original articles found in specialized literature (Akshita et al., 2020; Franco et al., 2020; Jin et al., 2020; Ma et al., 2020; Sacco et al., 2020; Zhang et al., 2021; Shitrit et al., 2020; Su et al., 2020; Vuong et al., 2020; Wenhao et al., 2020; Zhang et al., 2020; Bai et al., 2021; Hattori et al., 2021; Isgrò et al., 2021; Liu C. et al., 2021; Mody et al., 2021; Rothan and Teoh, 2021; Liu H. et al., 2021), and in-house acquired data from our group (including experimental results for the *in silico* hits selected from our first virtual screening campaign). The literature search and data compilation from the COVID Moonshot database were performed in February 2021. Compounds with IC_50_ < 10 µM or with a percentage of enzyme inhibition >50% at 10 µM were labelled as ACTIVE. In contrast, compounds with IC_50_ > 20 μM, percentage inhibition <80% at 20 µM or 50 μM, or percentage enzyme inhibition <50% at 10 µM were labelled as INACTIVE. The dataset compounds, represented in SMILES format, were standardized through an in-house script using the MolVS package. This in-house script is available upon request to the corresponding authors. Duplicate data and compounds with inconsistent labels from different sources were excluded. Finally, since only 0D-2D molecular descriptors were used for modelling purposes when data associated with different optical isomers were reported, only one of them was retained whenever both isomers belonged to the same activity class, and the compounds were disregarded if the isomers belonged to different activity classes. In total, 134 active and 281 inactive compounds remained in the curated dataset. Note that all compounds used as training examples in this second virtual screening (VS) campaign were either extracted from peer-reviewed sources (287 compounds) or from internal screening under standardized conditions (128 compounds). This has possibly resulted in more reliable and less noisy data, as discussed later. The heatmap included in Supplementary Material (Supplementary Figure S1) shows the molecular diversity of the dataset. The dataset, along with activity class for each compound, is provided as Supplementary Material, in. csv format (Data Sheet 1.csv).

### 2.2 Dataset splitting into training, test and validation sets

#### 2.2.1 First ligand-based modelling

The dataset was representatively divided into three different sets: a training set, used to train QSAR classifiers; a validation set, used for the validation of the individual QSAR models and to select which models (and how) would be combined into a model ensemble; and a test set, used to assess the performance of the model ensembles. The validation and test sets were complemented by decoys to provide retrospective screening sets 1 and 2, as described in Section 2.4.1. The representative partitioning of the dataset was generated using a serial combination of clustering procedures. First, we used the hierarchical clustering method included in LibraryMCS software (version 17.2.13.0–ChemAxon), which relies on the Maximum Common Substructure (MCS). From the resulting clusters, a compound from each cluster was randomly chosen and used as a seed to perform non-hierarchical clustering using the k-means algorithm, as implemented in the Statistica 10 Cluster Analysis module. Such procedure was performed in an independent manner for the ACTIVE and INACTIVE categories.

#### 2.2.2 Second ligand-based modelling

The dataset was representatively divided into three different sets: a training set, used to train QSAR classifiers; a validation set, used for the validation of the individual QSAR models and to select which models (and how) would be combined into a model ensemble; and a test set, used to assess the performance of model ensembles. The validation and test sets were complemented by decoys to provide retrospective screening sets 1 and 2, respectively, as described in Section 2.4.2. To sample the dataset representatively, we used the iterative Random subspace Principal Component Analysis (iRaPCA) clustering (Prada Gori et al., 2022a), an iterative in-house clustering algorithm based on feature bagging, dimensionality reduction and the k-means algorithm, which provides almost optimal performance in benchmark exercises (Prada Gori et al., 2022a; 2022b). Compounds from the ACTIVE and INACTIVE categories were clustered separately.

### 2.3 Molecular descriptor calculation, modelling procedure and model validation

#### 2.3.1 First ligand-based modelling

A total of 3668 conformation-independent descriptors were computed using Dragon 6.0. Using the random subspace approach (Yu et al., 2012; El Habib Daho and Chikh, 2015) 1,000 subsets of 200 descriptors each were obtained. A dummy dependent variable was then introduced, which took a value of 1 for compounds within the ACTIVE class and a value of 0 for compounds belonging to the INACTIVE class. 1,000 linear classifiers, one per subset, were obtained using a Forward Stepwise procedure. A maximum of eight descriptors per model were allowed to avoid overfitting. In addition, a maximum Variance Inflation Factor (VIF) of 2 was tolerated. No descriptor with a regression coefficient with a *p*-value above 0.05 was allowed into the model. The R environment was used for all data analyses. The R package data table (https://cran.r-project.org/package=data.table) was used to process the datasets.

#### 2.3.2 Second ligand-based modelling

A total of 1613 conformation-independent descriptors were computed using the Mordred package (Moriwaki et al., 2018). Descriptors with a variance below 0.05 across the training set were excluded from the descriptor pool. The random subspace approach (Yu et al., 2012; El Habib Daho and Chikh, 2015) was applied to the remaining descriptors to obtain 1,000 subsets of 200 descriptors each. Highly correlated descriptors (Pearson’s correlation >0.85) were not allowed within a given subset. A dummy dependent variable was then introduced, which took a value of 1 for compounds within the ACTIVE class and 0 for compounds belonging to the INACTIVE class. 1,000 linear classifiers, one per subset, were obtained using a Forward Stepwise procedure. A maximum of 16 descriptors per model were allowed to avoid overfitting.

The probability of spurious correlations and the robustness of the models were assessed using Fisher’s randomization and Leave-Group-Out (LGO) cross-validation, respectively. In each LGO round, randomly stratified subsets of 10% of the total training set samples were removed from the training set. A total of 500 randomizations and 500 LGO folds were considered. The results for both internal validation tests were reported as the average accuracy across 500 rounds and compared with the accuracy of the model inferred from the original training set, as well as the No-Model error rate (NOMER) (Gramatica, 2013). The predictive ability of each model was further assessed using external validation.

### 2.4 Retrospective screening experiments

#### 2.4.1 First ligand-based modelling

To estimate the enrichment performance of the models from the first screening campaign in a realistic setting, two retrospective VS experiments were conducted. The first retrospective screening was performed by seeding the active compounds of the validation and the test sets among a high number of decoys generated through the Directory of Useful Decoys enhanced (DUD-e, Mysinger et al., 2012). Different enrichment metrics have been calculated to assess the enrichment behavior of the models: the Area Under the Receiver Operating Characteristic curve (AUC ROC), the Boltzmann-Enhanced Discrimination of ROC (BEDROC), the Area Under the Precision Recall curve (AUPR), and the Enrichment Factor in the top-ranked 1% (EF_0.01_) (Truchon and Bayly, 2007; Saito and Rehmsmeier, 2015). The best-performing individual models in this first screening were combined as described in the following subsection, and the performance of the resulting ensembles was assessed through a second retrospective screen, where the active compounds of retrospective screening set 1 were seeded among a high number of decoys, also obtained via DUD-e.

#### 2.4.2 Second ligand-based modelling

To estimate the enrichment performance of the models from the second screening campaign, two retrospective VS experiments were also conducted. The first retrospective screening was performed by seeding the validation set among a high number of decoys generated through the LIDEB’s Useful Decoys (LUDe) tool (Fallico et al., 2022). LUDe is an in-house method conceptually similar to DUD-e, but additional filters have been implemented to ensure the topological dissimilarity between the decoys and the active compounds that are used as queries, usually resulting in enhanced degree of embedding between the decoys and the queries, as well in reduction of the doppelganger score (Prada Gori et al., 2022b). Different enrichment metrics have been calculated to assess the enrichment behavior of the models: the Area Under the Receiver Operating Characteristic curve (AUC ROC), the Boltzmann-Enhanced Discrimination of ROC (BEDROC), the Area Under the Precision Recall curve (AUPR), and the Enrichment Factor in the top-ranked 1% (EF_0.01_) (Truchon and Bayly, 2007; Saito and Rehmsmeier, 2015). The best-performing individual models in this first screening were combined, as described in the following subsection, and the performance of the resulting ensembles was assessed through a second retrospective screen, where the test set was seeded among a large number of decoys, also obtained via LUDe. Since normality and/or equal variances assumptions were not met by the enrichment metrics used, the performances of the individual models and the best model ensemble were statistically compared using the Yuen-Welch test (Wilcox, 2012).

### 2.5 Ensemble learning

The combination of individual classifiers into meta-classifiers frequently provides better generalization and predictivity (Min, 2016; Hyun et al., 2020); we have thus selectively combined the best individual classifiers, based on their performance in the first retrospective screening. Four different combination schemes were tested: the average (AVE) and the minimum (MIN) score, the average ranking (RANK) provided by the model ensembles, and the average voting (VOT) as computed by Zhang and Muegge (Zhang and Muegge, 2006).

### 2.6 Molecular docking

As part of a previous investigation, we benchmarked three docking protocols for the SARS-CoV-2 MPro system: QuickVina2, AutoDock4-GPU, and AutoDock4 hydrated. Using the original dataset of 816 molecules compiled for the first VS campaign and a set of 52 SARS-CoV-2 MPro monomeric structures released before October 2020 retrieved from https://covid-19.bioreproducibility.org (a database of carefully curated and validated COVID-19 protein structures), we assessed the pose prediction and the VS accuracy of these protocols. Regarding the pose prediction, evaluated by means of re-docking and cross-docking experiments, all docking protocols were able to reproduce the experimental binding mode with only modest errors in terms of root-mean-square-deviation (RMSD). However, none of them was able to retrieve active compounds at the top positions of the ranking, as reflected by the poor enrichment metrics obtained.

The best-performing docking protocol in terms of pose prediction accuracy was AutoDock4-GPU, which yielded a mean RMSD of 0.955 ± 0.658 Å for re-docking and cross-docking simulations across the entire set of structures and ligands. Among them, the neutron diffraction crystal structure (PDB-ID: 7JUN) achieved one of the smallest RMSD values (0.914 ± 0.699 Å), calculated as the median of all ligands (Supplementary Figure S2). Based on these results, the 7JUN structure was selected to reevaluate the VS accuracy of the protocol using the refined dataset compiled for QSAR modelling (415) in this investigation. Some molecules in this refined dataset failed to pass our ligand preparation pipeline; therefore, they were excluded. Thus, the final validation set (408) was comprised of 134 active and 274 inactive compounds. All docking conditions were the same as those previously described. Briefly, a grid box of 20 × 20 × 20 Å enclosing all crystallized ligands was defined with the default spacing of 0.375 Å. The grid maps were calculated using Autogrid, the number of energy evaluations and the local-search algorithm were set on-the-fly for each ligand based on a built-in heuristic, and the automatic stop criterion based on energy convergence was turned on. A total of 200 docking runs were performed for each ligand. All other parameters were set to default values.

Because of the good results obtained for pose prediction accuracy, the same docking protocol was used to predict and analyze the binding mode of representative structures selected during the second VS campaign with the ligand-based model and experimentally tested.

### 2.7 Prospective virtual screening

The model ensemble that showed the best performance in the second retrospective screen of each campaign was used in the VS of an in-house library of 6,266 chemical compounds (note: 2,895 of them available in solid state or in solution). The molecular representations of the compounds in each database were standardized as previously described for the datasets. The optimal cutoff value for the ensemble score was chosen through the analysis of Positive Predictive Value (PPV) surfaces (Bélgamo et al., 2020). As a final selection criterion, we assessed whether the *in silico* hits belonged to the applicability domain of the model, using the leverage approach (Yasri and Hartsough, 2001), where 3*d/n* is defined as the critical value, *d* is the number of descriptors included in each model and *n* is the number of training set compounds.

### 2.8 Chemical compounds

The compounds were provided by a large consortium of chemistry groups that co-authored this study. For the majority of the compounds, details about their synthesis and characterization can be found in the following references (Hayashi et al., 1980; Mallet et al., 1993; Zagotto et al., 1993; Agnihotri et al., 2005; Agnihotri and Misra, 2005; Crich and Vinod, 2005; Bohn et al., 2006; Bohn et al., 2007; Porcal et al., 2007; Chilin et al., 2008; Ducray et al., 2008; Castro et al., 2009; Mong et al., 2009; Zhang et al., 2009; Sato et al., 2010; Spampinato et al., 2010; Basu et al., 2011; Colombo et al., 2011; Tatina et al., 2012; Benítez et al., 2016; Ribaudo et al., 2016; Jäger et al., 2019; Posada and Serra, 2019; Posada et al., 2020; Zhu and Xie, 2020; Irabuena et al., 2022; Istifli et al., 2022; Posada et al., 2022; Colobbio et al., 2023) or will be published elsewhere.

### 2.9 Expression and purification of SARS-CoV-2 MPro

The recombinant form of SARS-CoV-2 MPro was expressed and purified as essentially described in Zhang et al., 2020, except that the fractions eluted from the Mono Q column containing recombinant protein with high purity were pooled and subjected to buffer exchange (20 mM Tris, 150 mM NaCl, 1 mM EDTA, 1 mM DTT, pH 7.8) using a PD 10 desalting column.

### 2.10 MPro activity assay

MPro activity was determined by de-quenching of Edans fluorescence (5-((2-Aminoethyl)amino)naphthalene-1-sulfonic acid) upon proteolytic cleavage of a synthetic peptide (Dabcyl-KTSAVLQ↓SGFRKM-E (Edans)-NH2; United Biosystems-USA). The assay was performed in a 96-well black microplate (total assay volume 200 μL) and read using a Varioskan Lux microplate reader (Ex/Em = 340 nm/490 nm). Different parameters were routinely controlled for validating the assay (signal to background ratio >7, Z′ factor >0.75, and relative fluorescence units >10). All samples were analyzed at least in duplicate.

For the screening of the chemolibrary, the compounds (assay concentration: 10 or 25 μM, freshly prepared from solid or from stock solutions in 100% v/v DMSO stored at −20°C) were incubated with MPro (90 nM) in reaction buffer (Tris 20 mM, pH 7.8, 150 mM NaCl, 1 mM EDTA, 5% v/v DMSO) for 60 min at 25°C. The peptidic substrate (5 μM) was then added and fluorescence monitored for at least 30 min. Blank (reaction buffer + substrate), full-activity (MPro + substrate) and inhibition (MPro treated with 25 μM ebselen or iodoacetamide + substrate) controls were run in parallel. Drugs that inhibited MPro activity ≥50% under such conditions were considered hits and their IC_50_ values determined by measuring enzyme activity at different compound concentrations (7–8 points concentrations prepared in serial dilutions) and under the conditions described above. The data were fitted to the best linear or nonlinear equations using GraphPad Prism Software (version 6.0) to obtain the IC_50_.

### 2.11 Cytotoxicity assays in human-derived cell lines

Cytotoxicity against the human lung cell line A549 (ATCC CCL-185™) was determined for the most potent MPro inhibitors using the WST-1 Cell Proliferation Reagent (Roche). A549 cells were grown in DMEM (Gibco) supplemented with 10% v/v fetal bovine serum (FBS; GIBCO) at 37°C in an atmosphere of 5% CO_2_. Cells with no more than 12 passages were used in the cytotoxicity assays. To assess cell viability, 100 µL of a cell suspension (2 × 10^4^ cells/well) were seeded in a 96-well cell culture plate and incubated overnight at 37°C and a 5% CO_2_ atmosphere. Next, the compounds dissolved in culture medium with 0.5% v/v DMSO were added at different concentrations to the wells (100 µL/well) with three replicates each and incubation extended for additional 24 h. Control wells included untreated cells (cytotoxicity negative control), cells treated with 0.1% Triton X-100 (cytotoxicity positive control) and cells treated with 0.5% DMSO (vehicle control). After incubation, WST-1 (Roche) diluted 1:10 in culture medium was added (100 µL/well) and the culture plate was incubated for 1 h at 37°C. Absorbance was measured at 450 nm in a microplate reader. The cytotoxicity of each compound was expressed as percentage of cell viability normalized to controls.

### 2.12 SARS-CoV-2 cell infection assay

The antiviral activity of the MPro hits was determined using a 384-wells microplate fluorescent-based cell infection assay for SARS-CoV-2 (Jeon et al., 2020). The experiments were performed in compliance with the guidelines of the Korean National Institutes of Health, using enhanced Biosafety Level 3 (BSL-3) containment procedures in laboratories approved for use by the Korea Disease Control and Prevention Agency (KDCA). Briefly, Vero cells were sourced from ATCC (CCL-81) and grown in DMEM (Welgene) supplemented with 10% v/v FBS and 1X Antibiotic-Antimycotic solution (Gibco) at 37°C and a 5% CO_2_ atmosphere. Vero cells were seeded at 1.2 × 10^4^ cells/well in DMEM, supplemented with 2% v/v FBS and 1X Antibiotic-Antimycotic solution in black 384-well, μClear plates (Greiner Bio-One), 24 h prior to the experiment. Then, the compounds or reference drugs (ten-point concentrations) and SARS-CoV-2 (βCoV/KOR/KCDC03/2020; MOI = 0.0125) were added to the wells and incubation extended for additional 24 h. Chloroquine diphosphate (Sigma), Remdesivir (MedChemExpress) and Lopinavir (Selleckchem) were used as the reference drugs. After 24 h of incubation, the cells were fixed and analyzed by immunofluorescence using an anti-SARS-CoV-2 nucleocapsid (N) protein antibody (Sino Biological Inc.) and an Alexa Fluor 488 goat anti-rabbit IgG (H + L) secondary antibody. The cell nuclei were stained with Hoechst 33,342 (Molecular Probes). Fluorescence microscopy images were taken with an Operetta CLS (PerkinElmer) and analyzed using Columbus™ (PerkinElmer) to quantify cell numbers and infection ratios. Antiviral activity was normalized to positive (mock no virus with 0.5% v/v DMSO) and negative (virus with 0.5% v/v DMSO) controls in each assay plate. IC_50_ values were calculated from data fit to sigmoidal equations using XLfit (Version 5.5) or GraphPad Prism Software (version 8). The quality of each assay was controlled by the Z′-factor and the coefficient of variation in percent (%CV).

## 3 Results

### 3.1 Ligand-based modelling

#### 3.1.1 First ligand-based modelling campaign

Table 1 shows the composition (in terms of active and inactive compounds) of the training, validation and test sets and how the validation and test sets were enriched with putative inactive compounds to provide, respectively, the chemical libraries used in retrospective screen 1 (to validate the enrichment performance of individual models and train the model ensembles) and retrospective screening 2 (to validate the enrichment performance of the model ensembles).

**TABLE 1:** Active and inactive compound composition of the training, validation, and test sets and both retrospective screening sets in the first VS campaign. The validation and test sets were expanded with decoys from DUD-e to obtain the retrospective screening sets 1 and 2; the final ratio of active compounds in each retrospective screening sets (Ya) is also provided in the table.

Dataset	Active	True inactive	Putative inactive or decoys	Ya
Training	42	42	-	0.5
Validation	17	348	850	0.0142
Test	17	348	850	0.0142

1,000 individual linear models (i.e., 1,000 individual classifiers) were generated from the training set by applying a combination of feature bagging and Forward Stepwise on a pool of 3,668 Dragon molecular descriptors. The individual classifiers were validated both internally and externally, initially employing a score cutoff value of 0.5 to discriminate between active and inactive compounds. The accuracy (Acc) over the training set and the internal validation results for the five best individual classifiers are summarized in Table 2. The five best individual models and the meaning of their molecular descriptors have been included as Supplementary Material. It is obvious that the models are not particularly robust, and on that basis, we decided to proceed to selective ensemble learning.

**TABLE 2:** Accuracy over the training set and cross-validation of the top five individual models in the first retrospective screening. In the case of the cross-validation and randomization tests, the mean accuracy across 500 rounds is provided; the standard deviation of the mean is presented in parentheses. The models were ordered according to their performance in the first retrospective screening.

Model	Acc (training set)	Mean Acc (cross-validation)
MODEL 324	0.845	0.650 (0.153)
MODEL 644	0.798	0.581 (0.163)
MODEL 739	0.774	0.655 (0.159)
MODEL 510	0.845	0.682 (0.148)
MODEL 390	0.857	0.678 (0.148)

**FIGURE 1:**
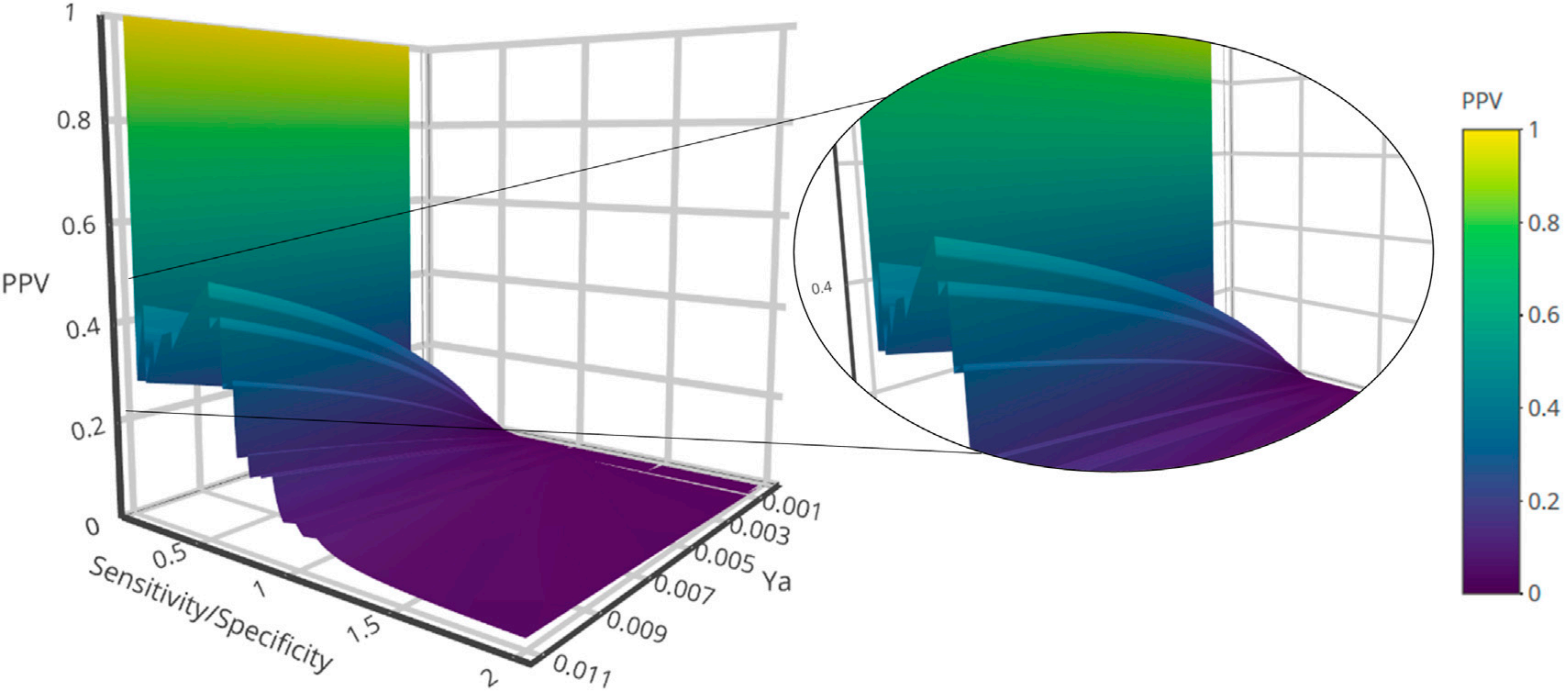
PPV surface from the first retrospective *in silico* screening against MPro (First ligand-based modelling campaign).

The systematic combination of the 2 to 100 individual models that showed the best performance in the first retrospective screening was performed using four different operators to combine the scores of the individual models comprising the ensemble. The model ensemble obtained by combining 50 models via the MIN operator (MIN-50) provided the best results across different metrics, greatly improving early and overall enrichment. The results in both retrospective screening experiments are shown in Table 3; for comparative purposes, the results of the best individual model (MODEL 324) are also included. A PPV surface for the first retrospective screening using MIN-50 was generated to select an optimal cutoff for the prospective VS (Figure 1).

**TABLE 3:** Performance of the best individual model and the best model ensemble in retrospective screening experiments. Standard deviations of the enrichment metrics (obtained using bootstrapping) are presented within parentheses.

Model	Retrospective screen	AUCROC	BEDROC (*α* = 100)	AUPR	EF_0.01_
MODEL 324	1	0.883 (0.013)	0.017 (0.007)	0.068 (0.007)	0
	2	0.878 (0.017)	0.061 (0.018)	0.092 (0.013)	0
MIN-50	1	0.930^a^ (0.029)	0.533^a^ (0.063)	0.406^a^ (0.067)	33.98^a^ (6.29)
	2	0.905^a^ (0.019)	0.438^a^ (0.073)	0.277^a^ (0.066)	32.47^a^ (6.60)

^a^ Statistically different from the best individual model on the same chemical library, *p* < 0.05.

#### 3.1.2 Second ligand-based modelling campaign

Table 4 shows the composition of the training, test, and validation sets used in the second modelling and VS campaign (including the composition of each in terms of active and inactive compounds), and how the test and validation sets were enriched with putative inactive compounds from LUDe to provide the chemical libraries used for retrospective screening 1 and retrospective screening 2.

**TABLE 4:** Composition of active and inactive compounds in the training, validation and test sets used for model training and retrospective screening in the second VS campaign. The validation and test sets were expanded with LUDe decoys to provide chemical libraries to be used in retrospective screenings 1 and 2, respectively. The final ratio of active compounds in each retrospective screening sets (Ya) is also provided in the table.

Dataset	Active	True inactive	Putative inactive or decoys	Ya
Training	80	80	-	0.5
Validation	27	101	1446	0.0174
Test	27	100	1430	0.0176

Again, 1,000 individual linear models were generated from the corresponding training set by applying a combination of feature bagging and Forward Stepwise on a pool of 1,613 Mordred molecular descriptors. The individual classifiers were validated both internally and externally, initially using a score cutoff value of 0.5 to discriminate between active and inactive compounds. The internal validation results for the five best individual classifiers, according to their AUC ROC in the first retrospective screening, are summarized in Table 5. The five best individual models and their molecular descriptors have been included in the Supplementary Material. It can be observed from the results of the randomization test that the chance of spurious correlations between the dependent and independent variables is rather low, and the accuracy of the randomized models is invariably similar to 0.5, as expected. In contrast, the results of the cross-validation experiments indicate some degree of overfitting (systematically, the mean accuracy across the cross-validation folds is below the accuracy in the training set, for the five models).

**TABLE 5:** Internal validation of the top five individual models in the first retrospective screening. In the case of the cross-validation and randomization tests, the mean accuracy across 500 rounds is provided, and the standard deviation of the mean is presented in parentheses. The models were ordered according to their performance in the first retrospective screen.

Model	Acc (training set)	Mean Acc (cross-validation)	Mean Acc (randomization)
MODEL 25	0.825	0.714 (0.102)	0.501 (0.106)
MODEL 361	0.813	0.701 (0.109)	0.496 (0.095)
MODEL 77	0.819	0.705 (0.115)	0.498 (0.099)
MODEL 273	0.831	0.730 (0.104)	0.500 (0.094)
MODEL 442	0.894	0.673 (0.115)	0.500 (0.085)

Owing to the suboptimal results of the individual classifiers in the cross-validation, we used ensemble learning to improve robustness. The performance of the individual models and the model ensembles was comparatively assessed in two retrospective screening campaigns, where known MPro inhibitors were seeded among (known and putative) non-inhibitors. The best individual model displayed an AUC ROC of 0.934 ± 0.007, a BEDROC of 0.274 ± 0.057, an AUPR of 0.221 ± 0.038 and an EF_0.01_ of 0.29 ± 0.07 in the first retrospective screen, indicating that there was plenty room for improvement (note that, despite the good AUC ROC, the early enrichment metrics clearly exhibit suboptimal values).

**FIGURE 2:**
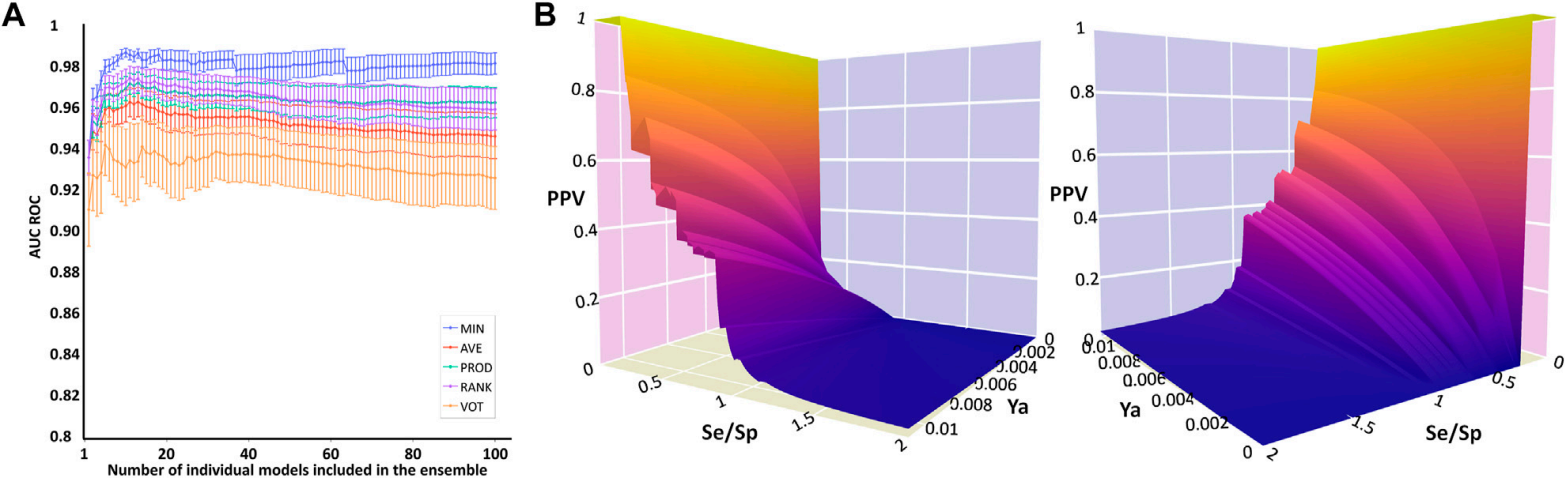
Data plots from retrospective and prospective *in silico* screening against MPro. **(A)** AUC ROC obtained in the retrospective screening as a function of the number of combined models for each operator. **(B)** Two different views of the PPV surface of the MIN-22 ensemble.

The systematic combination of the 2 to 100 individual models that showed the best performance in the first retrospective screening was performed, using four different operators to combine the individual models’ scores (Figure 2A). The model ensemble obtained by combining 22 models via the MIN operator (MIN-22) provided the best results across different metrics, greatly improving early and overall enrichment. The results in the two retrospective screenings are shown in Table 6; for comparative purposes, the results of the best individual model (MODEL 25) have also been included.

**TABLE 6:** Performance of the best individual model and the best model ensemble in retrospective screening experiments. Standard deviations of the enrichment metrics (obtained through bootstrapping) are presented in parentheses.

Model	Retrospective screen	AUCROC	BEDROC (*α* = 100)	AUPR	EF_0.01_
MODEL 25	1	0.934 (0.007)	0.274 (0.057)	0.221 (0.038)	0.29 (0.07)
	2	0.837 (0.025)	0.115 (0.027)	0.101 (0.017)	0.04 (0.04)
MIN-22	1	0.982^a^ (0.04)	0.739^a^ (0.039)	0.663^a^ (0.044)	42.45^a^ (4.38)
	2	0.900^a^ (0.025)	0.614^a^ (0.051)	0.489^a^ (0.052)	38.42^a^ (4.32)

^a^ Statistically different from the best individual model on the same chemical library, *p* < 0.001.

### 3.2 Prospective virtual screening campaigns and experimental validation

#### 3.2.1 First screening campaign

By analyzing the PPV surfaces (Figure 1) built upon the first retrospective screen, an optimized score cutoff value of 0.242 was chosen for the MIN-50 ensemble to identify *in silico* hits, corresponding to an estimated specificity of 0.981 and a minimum PPV value of 0.253 for a hypothetical yield of active compounds (Ya) of 1%. This suggests that, if there is one active compound per 100 compounds in the screened chemical library, one every four *in silico* hits is theoretically expected to confirm the prediction when submitted to experimental confirmation. If a Ya of 0.1% was assumed, the same score cutoff value would determine a minimum PPV of 0.04, meaning that more than least 1 in 25 *in silico* hits would theoretically confirm the predicted activity.

The MIN-50 was applied in the VS of our in-house library comprising 6,266 compounds. The ligand-based model ensemble identified 83 *in silico* hits from the in-house database, 18 of which were also chosen by structure-based screening. The identities of these *in silico* hits are shown in Supplementary Material in. csv format (Data sheet 2.csv). Based on compound availability, 46 of these hits, belonging to 11 different families, and 100 closely related derivatives were subjected to experimental screening (see Supplementary Table S1).

The screening assay was adapted to favour the detection of weak competitive or slow-binding inhibitors by pre-incubating the compound (10 μM) with MPro (90 nM) for 60 min prior to the addition of the fluorogenic substrate at a sub-*K*_M_ concentration (5 μM). None of the tested molecules fulfilled the hit criteria: MPro activity ≤50% at 10 μM.

Next, several virtual hits (34) were further tested at a 2.5-fold higher concentration (25 μM; see Supplementary Table S1). As shown in Table 7 and Supplementary Table S1, none of the *in silico* hit candidates was able to significantly inhibit MPro activity, which ruled out that the initial and more demanding hit criterion was responsible for the negative outcomes.

**TABLE 7:** Most active compounds targeting MPro identified during the 1^st^ round of screening.

Category	Compound code	Structure	MPro activity (%) at 25 μM or *IC*_*50*_ (μM)	VS^a^ hit
BBHPP^b^	1a	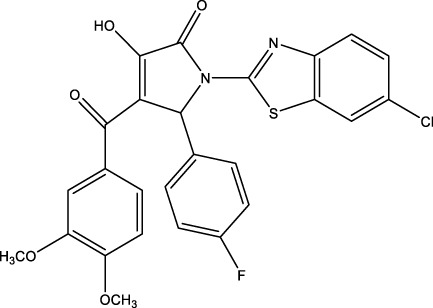	34.8 ± 3.4	No
PM^c^	2a	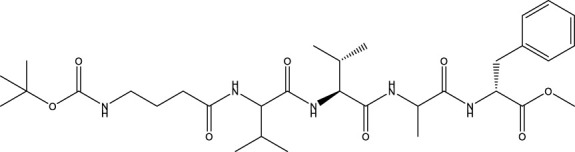	***32.1 ± 1.4*** ^d^	Yes
	3a	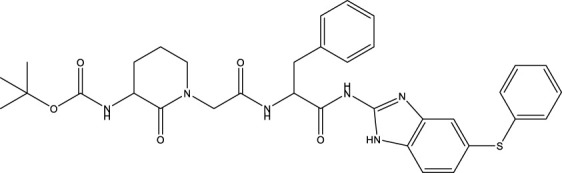	64.9 ± 11.6	Yes
QZ^e^	4a	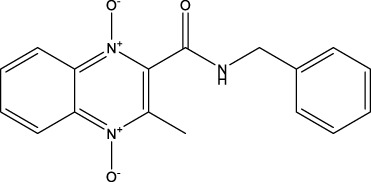	59.4 ± 3.6	No
SFH^f^	5a	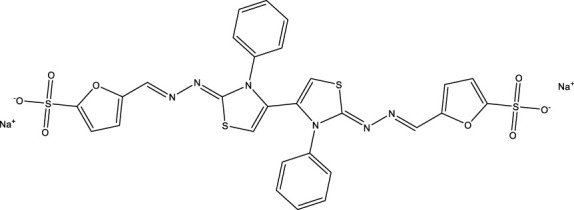	65.6 ± 10.9	Yes
G^g^	6a	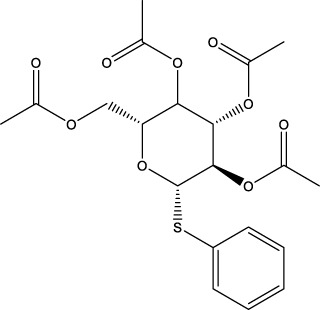	60.9 ± 1.5	No
	7a^h^	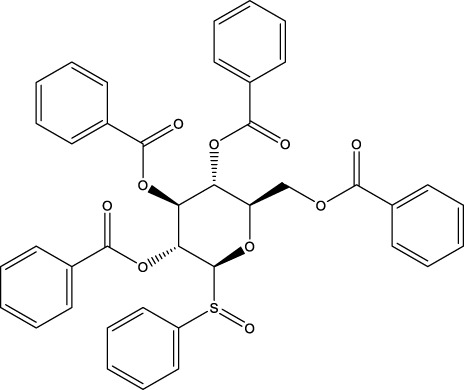	***20.2 ± 1.9*** ^d^	No
G^g^	8a	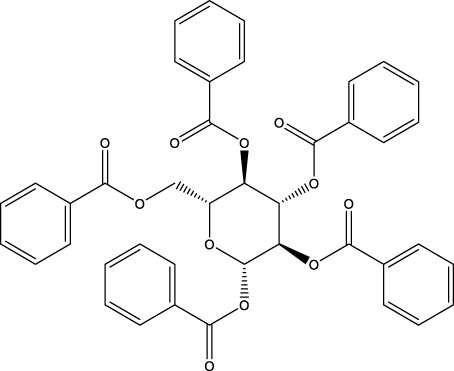	67.0 ± 2.5	No
F^i^	5b	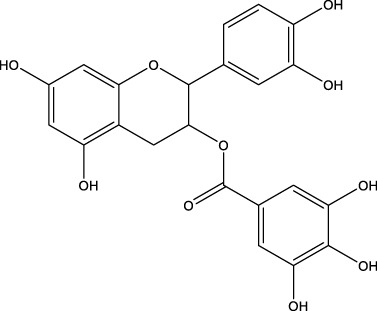	0.0 ± 0.3	No

^a^ VS: virtual screening.

^b^ BBHPP: 1-(benzo[d]thiazol-2-yl)-4-benzoyl-3-hydroxy-5-phenyl-1H-pyrrol-2(5H)-one.

^c^ PM: petidemimetic.

^d^ IC_50_ (μM).

^e^ QZ: quinazoline.

^f^ SFH: sulfonatofuran hydrazine bithiazole.

^g^ G: glycosides.

^h^ IC_50_ SARS-CoV-2 > 50 μM, CC_50_ Vero cells >50 μM.

^i^ F: flavonol.

**FIGURE 3:**
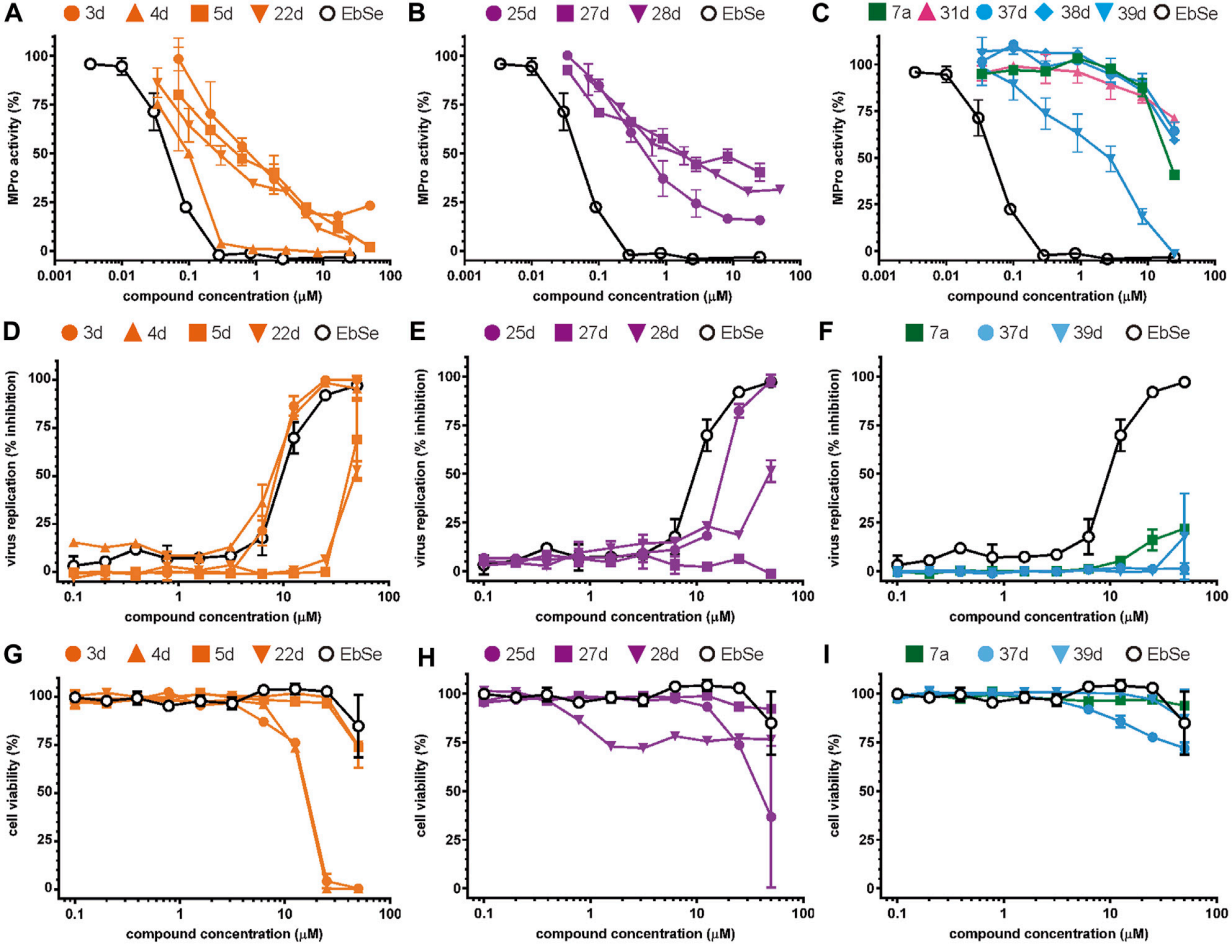
Concentration-dependent activity of the identified hits. The inhibitory activity of the selected compounds is shown for **(A–C)** MPro, **(D–F)** SARS-CoV-2 infected Vero cells, and **(G–I)** viability of Vero cells. In orange, the data for the benzofuroxan derivatives [plots **(A**, **D**, **G)**]; in violet, those corresponding to chalcones [plots **(B, E, H)**]; in green, a glycoside hit; in magenta, a sulphonamide hit; in light blue, different singletons [plots **(C, F, I)**]. All compounds were tested at ≥7 different concentrations (serial 1/3 or 1/2 dilutions for MPro assays or biological assays, respectively), at least in duplicate. Ebselen (EbSe) was included in all assays as positive control.

The screening was further extended to 44 analogues of the previously tested compounds. Among them, only one benzoyl-thiazol derivative (**1a**) and one glycoside molecule (a pyranose substituted with three benzyl and one phenylsulfinyl moiety, compound **7a**) resulted active against MPro (IC_50_ < 25 μM; Table 7; Figure 3C).

Regarding the 40 compounds unrelated to the VS hits, two set of molecules were tested: flavonoids (pure samples or partially purified extracts) and a family of synthetic pyrazoles. The former were chosen based on recent reports describing their anti-MPro (Jang et al., 2020) or anti-SARS-CoV-2 activity by blocking viral entry into host cells (Henss et al., 2021). The latter were selected considering the presence of this scaffold in several compounds with activity against different viruses and molecular targets thereof (reviewed in Khan et al., 2016). Interestingly, epicatechin gallate (**5b**) exerted full inhibition of MPro at 25 μM (Table 7), whereas the non-galloylated epicatechin and partially-purified flavonoid-enriched fractions from grapes showed a negligible activity against the viral protease (Supplementary Table S2). Of the 35 pyrazoles assayed, seven showed MPro inhibition in the range of 15%–30% at 10 or 25 μM, six appeared to stimulate MPro activity by more than 15% and the remaining ones proved inactive or interfered with the assay (Supplementary Table S2). In conclusion, none of the tested pyrazoles qualified as MPro hit.

The antiviral activity of epicatechin gallate (**5b**) was not investigated here but a recent study showed that the related analogue epigallocatechin gallate inhibited SARS-CoV-2 replication (IC_50_ = 1.73 μg/mL or 3.9 μM in a 72 h assay) (Henss et al., 2021). The bioactivity of the second MPro hit identified during the 1^st^ screening campaign, glycoside **7a**, was evaluated and proved to be weakly active against SARS-CoV-2 (15%–21% inhibition of viral replication at 25 μM and 50 μM, respectively; Figure 3F) and not cytotoxic to Vero cells (CC_50_ > 50 μM; Figure 3I).

Considering these results together, the hit ratio for the VS screening candidates from a large diversity of chemical families was null and increased to 1.4% when the experimental screening was extended to related molecules (two hits: **1a** and **7a**). In contrast, non-VS-guided experimental screening performed on 40 molecules belonging to two unrelated chemical families (i.e., flavonoids and pyrazoles) led to the detection of one hit (hit ratio: 2.5%), which was closely related to a molecule previously identified as MPro inhibitor (Jang et al., 2020). This finding supported the idea that the MPro ligand-based search algorithm developed had major predictive deficiencies.

Based on these disappointing results, the search algorithms were subjected to revision, fed with the new experimental information and with validated data obtained for empiric candidates (results to be published elsewhere), and the ensemble models and cut-off scores were optimized as in the first VS campaign.

#### 3.2.2 Second screening campaign

In this case, by analyzing PPV surfaces (Figure 2B) built upon the second retrospective screening experiment, an optimized score cutoff value of 0.546 was chosen for the MIN-22 ensemble to identify *in silico* hits, corresponding to an estimated specificity of 0.998, a minimum PPV value of 0.634 for a hypothetic Ya of 1% and of 0.147 for Ya = 0.1%. From these theoretical estimations, it can be observed that the ensemble of ligand-based models in this second VS campaign seems to significantly outperform the one used in the first campaign.

The MIN-22 was applied in the prospective VS of the in-house library, which yielded 43 MPro hit candidate molecules. The identities of these *in silico* hits are shown in Supplementary Material in. csv format (Data sheet 3.csv).

As expected from the strategy applied to optimize the search model, none of the new hit candidates resembled those from the 1st VS (Table 7; Supplementary Table S1). The new *in silico* hits can be grouped into four major families: benzofuroxans, chalcones, benzimidazol-2-yl-benzensulfonamides and furan-hydrazono-dihydrothiazoles (Tables 8–10) in addition to several singletons (Table 11). Constrained by compound availability, a total of 28 of the 43 predicted hits were assayed against MPro at a fixed concentration of 25 μM. The experimentally confirmed hit ratio for each compound family was of 60% for benzofuroxans, 50% for chalcones, 25% for benzimidazol-2-yl-benzensulfonamides, 0% for furan-hydrazono-dihydrothiazoles, and 12% for the singletons. Thus, on average, the confirmed hit ratio of the second VS campaign was of 29%, a value that largely surpasses the ratio obtained in the 1st screening (1.4%–2.5% for VS-related or -unrelated candidates).

**TABLE 8:** Benzofuroxan hits identified by the 2nd screening campaign. Compounds labelled or not with an asterisk correspond to *in silico* hits and structurally-related ones, respectively.

Compound code	Structure	MPro activity (%) or *IC*_*50*_ (μM)	IC_50_ SARS-CoV-2 (μM)	CC_50_ (μM) and *SI* ^a^	
				Vero cells	A549 cells
1d*	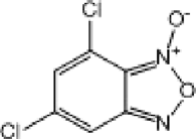	89.3 ± 1.9	ND^b^	ND	ND
2d*	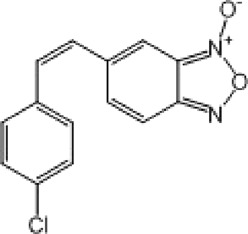	53.2 ± 0.2	ND	ND	ND
3d*	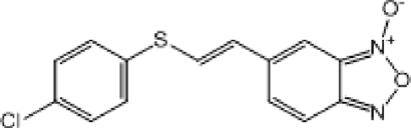	***0.73 ± 0.16*** ^c^	8.3	15.2 (*1.8*)^a^	<50
4d*	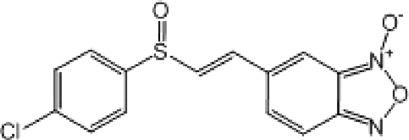	***0.121 ± 0.002*** ^c^	7.3	15.2 (*2.1*)^a^	50-100
5d*	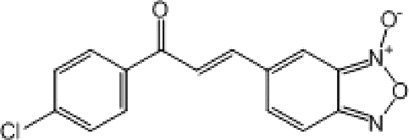	***0.64 ± 0.02*** ^c^	43.2	>50 (>*1.2*)^a^	>50
6d	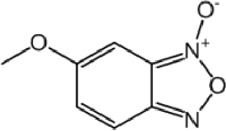	85.8 ± 3.5	ND	ND	ND
7d	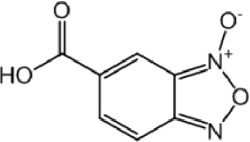	66.0 ± 4.0	ND	ND	ND
8d	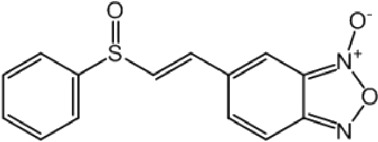	91.6 ± 0.1	ND	ND	ND
9d	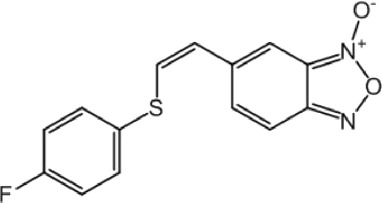	81.4 ± 21.5	ND	ND	ND
10d	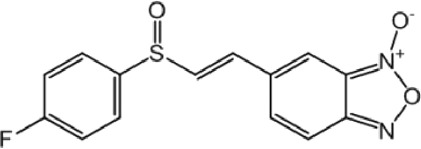	55.6 ± 3.4	ND	ND	ND
11d	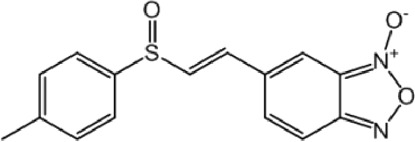	70.6 ± 0.8	ND	ND	ND
12d	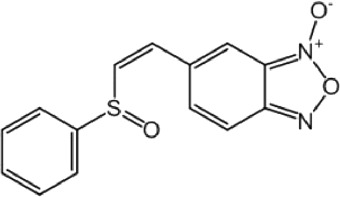	56.2 ± 0.5	ND	ND	ND
13d	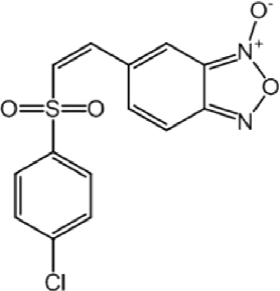	58.0 ± 0.3	ND	ND	ND
14d	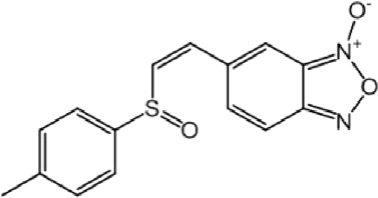	73.8 ± 4.4	ND	ND	ND
15d	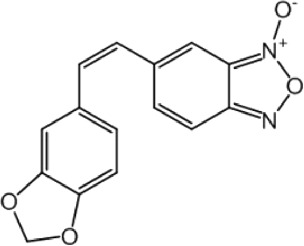	67.4 ± 1.8	ND	ND	ND
16d	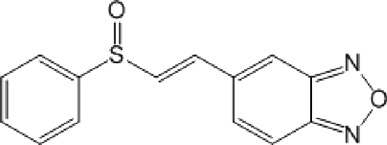	71.9 ± 11.0	ND	ND	ND
17d	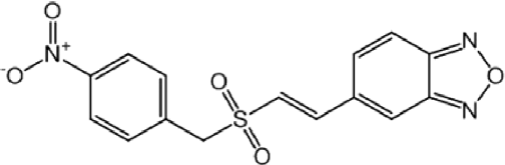	116.1 ± 15.0	ND	ND	ND
18d	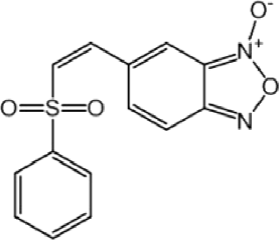	71.2 ± 0.9	ND	ND	ND
19d	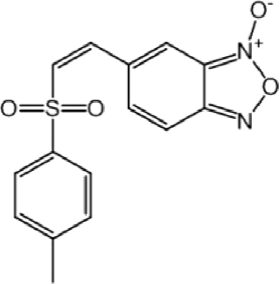	98.6 ± 2.4	ND	ND	ND
20d	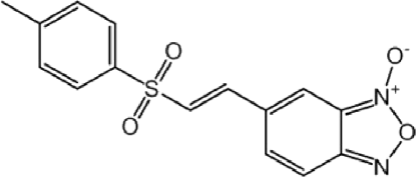	62.6 ± 4.9	ND	ND	ND
21d	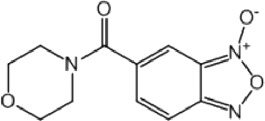	78.7 ± 3.2	ND	ND	ND
22d	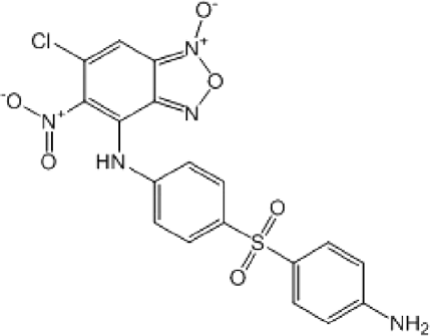	***0.37 ± 0.085*** ^c^	44.80	>50 (>*1.1*)^a^	>100
Control (Ebselen)	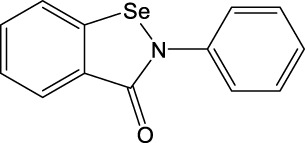	***0.049 ± 0.007*** ^c^	9.7	>50 (>*5.2*)^a^	>100
Remdesivir		ND	7.8	>50 (>6*.4*)^a^	ND
Chloroquine		ND	17.8	>150 (>8*.4*)^a^	ND
Lopinavir		ND	21.5	>150 (>2*.3*)^a^	ND

^a^ *SI*: selectivity index = CC_50_ mammalian cell/IC_50_ SARS-CoV-2.

^b^ ND: not determined.

^c^ IC_50_ (μM).

**TABLE 9:** Chalcone hits identified by the second screening campaign. All compounds shown in this table correspond to *in silico* hits.

Compound code (d)	Structure	MPro activity (%) or *IC*_*50*_ (μM)	IC_50_ SARS-CoV-2 (μM)	CC_50_ (μM) and *SI* ^a^	
				Vero cells	A549 cells
23	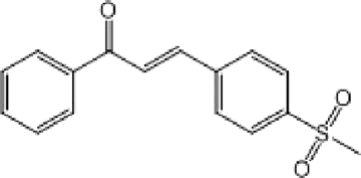	77.9 ± 5.6	ND^b^	ND	ND
24	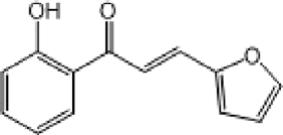	61.1 ± 4.4	ND	ND	ND
25	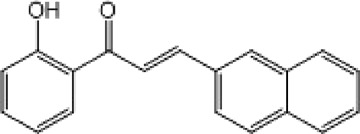	***0.46 ± 0.21*** ^c^	17.5	38.8 (>*2.2*)^a^	>50
26	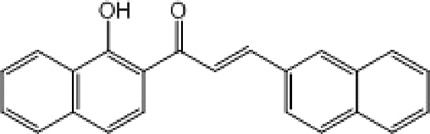	55.3 ± 1.4	ND	ND	ND
27	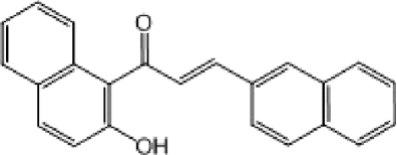	***5.76 ± 2.36*** ^c^	>50	>50 (∼*1*)^a^	>100
28	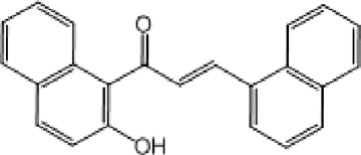	***1.35 ± 0.04*** ^c^	48.9	>50 (>*1*)^a^	>100

^a^ *SI*: selectivity index = CC_50_ mammalian cell/IC_50_ SARS-CoV-2.

^b^ ND: not determined.

^c^ IC_50_ (μM).

**TABLE 10:** Benzimidazol-2-yl-benzensulfonamides and furan-hydrazono-dihydrothiazole compounds. All compounds shown in this table correspond to *in silico* hits.

Category	Compound code (d)	Structure	MPro activity (%) or *IC*_*50*_ (μM)
Benzimidazol-2-yl-benzensulfonamides	29	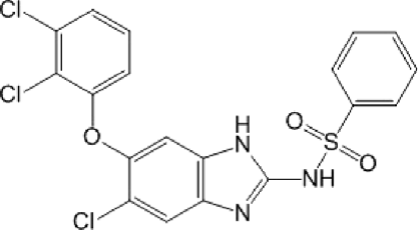	85.2 ± 4.1
	30	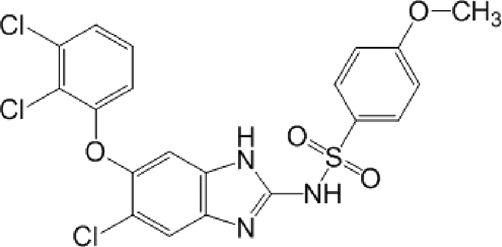	57.6 ± 4.7
	31	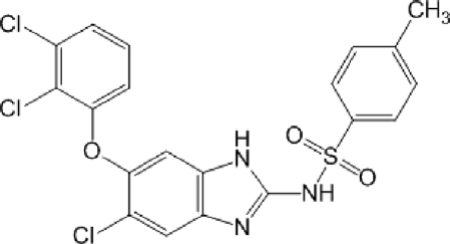	***> 25*** ^a^
	32	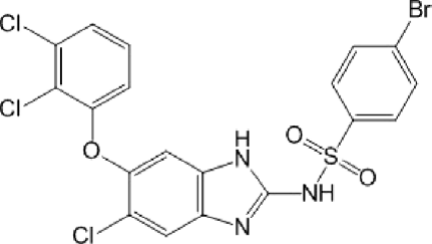	75.4 ± 6.5
Furan-hydrazono-dihydrothiazole	33	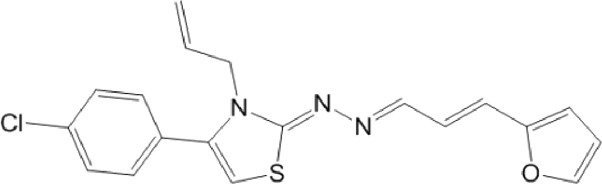	79.7 ± 4.6
	34	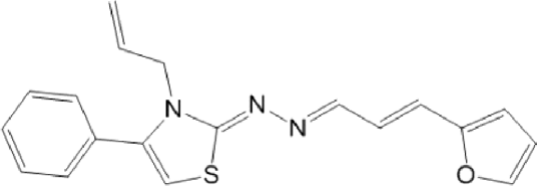	78.9 ± 3.4
	35	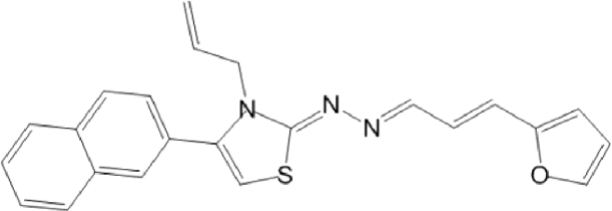	83.8 ± 3.4
	36	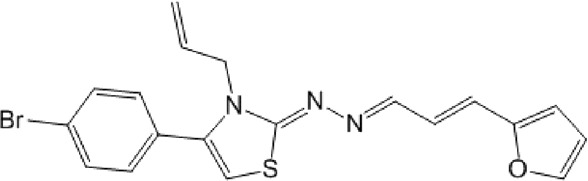	88.3 ± 4.5

^a^ IC_50_ (μM).

**TABLE 11:** Singleton compounds. All compounds shown in this table correspond to *in silico* hits.

Compound code (d)	Structure	MPro activity (%) or *IC*_*50*_ (μM)	IC_50_ SARS-CoV-2 (μM)	CC_50_ (μM) and *SI* ^a^	
				Vero cells	A549 cells
37	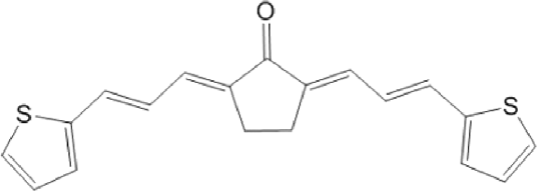	65.9 ± 5.0	>50	>50 (∼*1*)^a^	>100
38	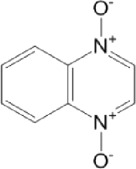	59.5 ± 0.9	ND^b^	ND	ND
39	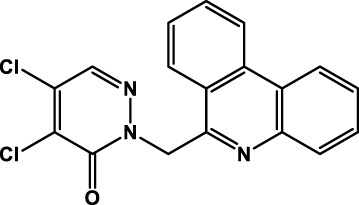	***1.97 ± 0.50*** ^c^	>50	>50 (∼*1*)^a^	50-100
40	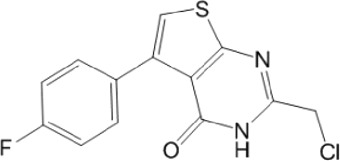	79.2 ± 4.3	ND	ND	ND
41	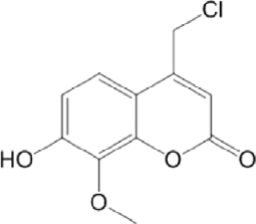	64.9 ± 4.3	ND	ND	ND
42	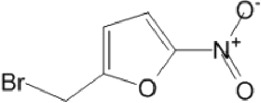	73.9 ± 0.0	ND	ND	ND
43	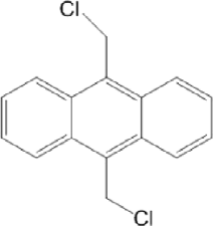	128.1 ± 0.8	ND	ND	ND
44	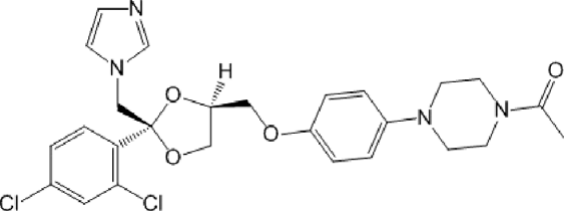	95.2 ± 0.4	ND	ND	ND
45	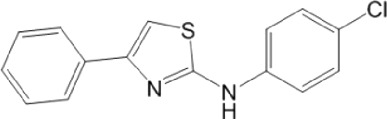	100.0 ± 2.2	ND	ND	ND

^a^ *SI*: selectivity index = CC_50_ mammalian cell/IC_50_ SARS-CoV-2.

^b^ ND: not determined.

^c^ IC_50_ (μM).

Most hits in the benzofuroxan series are monosubstituted with an alkyl moiety linked to different aryl or heterocyclic rings. The chemical nature of these groups appears to be determinant of the inhibitory potency towards the viral protease and the virus (Figures 3A, D). For instance, compounds **2d**-**5d** harbor a chlorobenzene group attached to the benzofuroxan moiety by different alkyl linkers with those having thiophenol α,β-unsaturated (**3d**), sulfoxide α,β-unsaturated (**4d**) and keto α,β-unsaturated (**5d**) groups being the most potent (three-digit nM IC_50_). A halogenated and nitrated benzofuroxan substituted with a sulfonyldianiline moiety (**22d**) showed a similar inhibitory activity against MPro. The thiophenol (**3d**) and sulfoxide (**4d**) analogues (IC_50_ vs. virus ∼8 μM) proved more active than **5d** and **22d** (IC_50_ vs*.* virus ∼44 μM) in inhibiting the proliferation of SARS-CoV-2. The antiviral potency of **3d** and **4d** was similar or 2-folds higher than that attained by the control drugs ebselen (IC_50_ vs. virus 10 μM) and Remdesivir (IC_50_ vs*.* virus 8 μM), or Chloroquine (IC_50_ vs. virus 18 μM) and Lopinavir (IC_50_ vs*.* virus 22 μM), respectively. However, compared to these drugs (SI from >2.3 to >8.4), the selectivity index of the benzofuroxans is marginal (SI > 1.1-2) when Vero cells are taken as host cell model (Figure 3G). Furthermore, **3d** and **4d** (CC_50_ ≤ 50 μM), but not **2d** and **22d** (CC_50_ > 50 and 100 >μM, respectively), displayed cytotoxicity against a human lung cell line (A549).

Among the chalcone series, those containing naphthalene groups (**25d**, **27d** and **28d**) were capable of inhibiting MPro with low to sub-μM IC_50_ values (0.5, 5.8 and 1.4 μM, respectively; Figure 3B). For these hits, the mono-naphthalene derivative (**25d**) was 3- to 12-folds more potent than those bi-substituted with a naphthalene or a naphthalenol moiety (**27d** and **28d**). Interestingly, the isomerism of the naphthalenol ring showed to be relevant for the anti-protease activity, since the isomer **27d** was 4-folds more active than **26d** (45% MPro inhibition at 25 μM). Furthermore, if the naphthyl group is connected to the rest of the molecule by an alpha-carbon atom (**28d**), instead of a beta carbon (**27d** or **26d**), the anti-MPro activity increases by 4- or 19-folds, respectively. With respect to the anti-SARS-CoV-2 activity, the most potent chalcone against MPro (**25d**) also proved to be the most active against the virus (IC_50_ vs virus 39 μM), though more than 2-folds less active than the control drugs (Table 8). In contrast, **27d** lacked antiviral activity whereas the analogue **28d** displayed activity against SARS-CoV-2 (18%–50% inhibition) at concentrations above 12.5 μM that also impaired cell viability by ∼25% (Figures 3E, H).

None of the benzimidazol-2-yl-benzensulfonamides (**29d**-**32d**) and furan-hydrazono-dihydrothiazoles (**33d**-**36d**) rated as *in silico* MPro hits met this expectation at the experimental level (Table 10) but exhibited moderate (25% and 42% inhibition for **32d** and **30d**, respectively; Figure 3C) or low (12%–20% for the remaining molecules) inhibitory activity against the protease when tested at 25 μM.

With respect to the singletons (Table 11; Figure 3C), only one compound (**39d**) was experimentally confirmed as MPro hit with an IC_50_ of 2 μM. Five of the singletons (**37d**, **38d**, **40d**-**42d**) showed a moderate inhibitory activity of MPro (protease inhibition between 20% and 40%) whereas three lacked activity against the protease (**43d**-**45d**). The antiviral activity was evaluated for two singletons, namely, **39d** and **37d**, and proved to be null (Figure 3F). In the case of **39d**, the apparent inhibition of SARS-CoV-2 replication observed at 50 μM (18% inhibition; Figure 3F) can be ascribed to the cytotoxic effect the compound exerted on the host cell (13% impairment of cell viability; Figure 3I).

### 3.3 Molecular docking

During target-based VS, a database of compounds is docked into the 3D structure/s of the target, and sorted according to their predicted binding energy. The algorithms available for pose generation and the scoring functions are predefined in each docking software, so the success of a target-based VS in terms of pose and score prediction highly depends on the software/algorithms selected and the system under study.

In the case of SARS-CoV-2 MPro, the VS accuracy of the docking-based model did not improve compared to our previous validation (AUCROC: 0.484 ± 0.029). This is in line with similar findings emerging from other investigations, where most docking protocols failed to correctly discriminate between experimentally confirmed active and inactive compounds, suggesting an intrinsic limitation of the methodology for this particular system, rather than a data quality issue (Alves et al., 2021; Llanos et al., 2021; Zev et al., 2021; Macip et al., 2022).

Conversely, as previously mentioned, the software has the ability to accurately reproduce the experimental conformation of the ligands in the MPro binding site (pose prediction accuracy); therefore, the interactions between the target and representative active and inactive compounds reported in this investigation were simulated.

**FIGURE 4:**
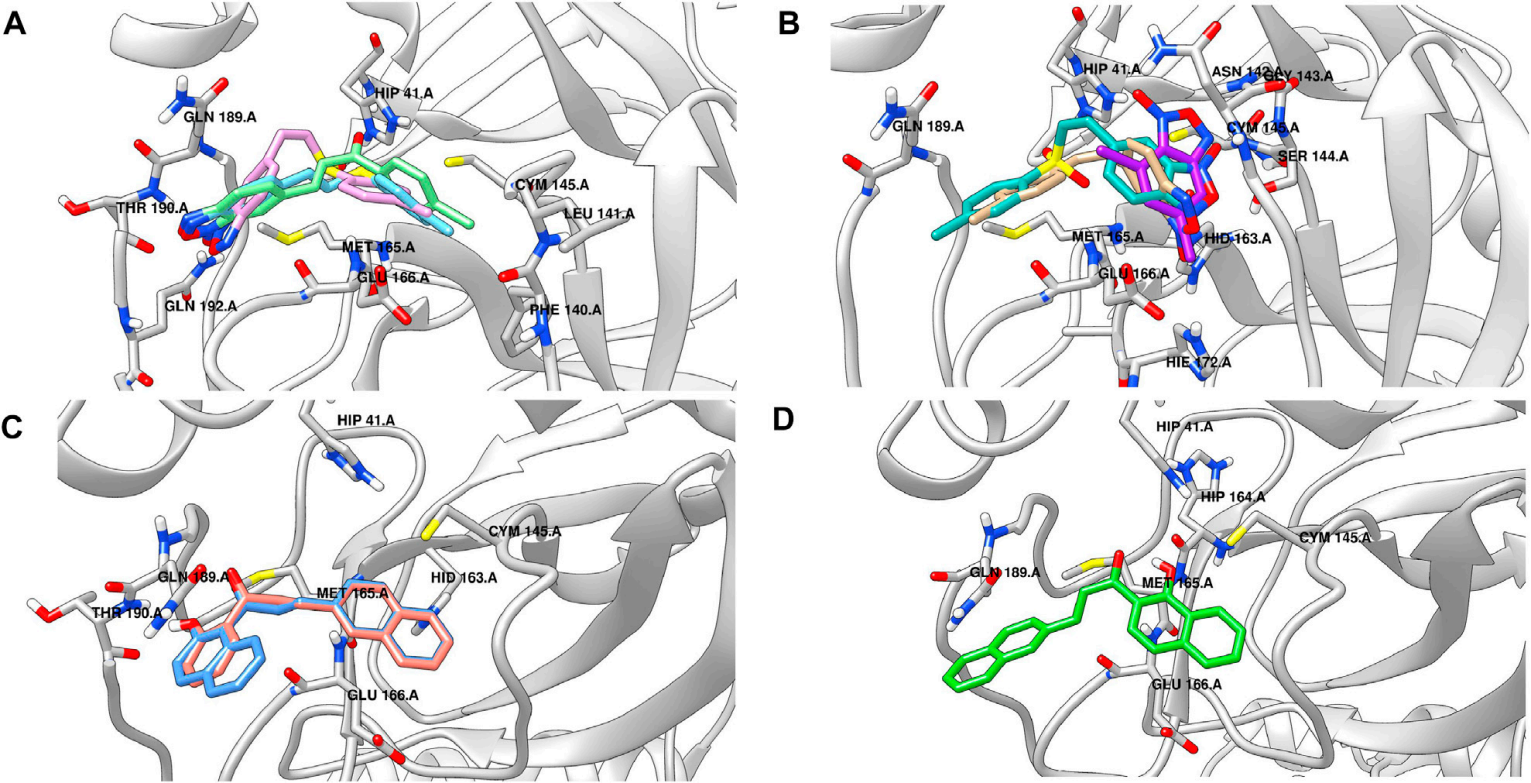
Binding poses predicted by docking for some representative active and inactive compounds reported herein. **(A)** Most active compounds of the benzofuroxan family: **3d** (sky blue), **19d** (plum), and **5d** (light green) **(B)** Less active compounds of the benzofuroxan family: **1d** (purple), **2d** (tan), and **19d** (light sea green). **(C)** Active compounds of the chalcone-related structures: **25d** (salmon) and **27d** (cornflower blue). **(D)** Less active compound of the chalcone-related structures: **26d** (lime green), an isomer of **27d**.

Figure 4A shows the binding poses predicted for the most active compounds of the benzofuroxan family. Compounds **3d**, **4d**, and **5d** exhibited the same orientation in the active site, promoting polar and hydrogen-bonding interactions between the heteroatoms of the oxadiazole-1-oxide ring and residues located in the loop of the S3 region (THR190 and GLN192), whereas the chlorophenyl substituents were located into the S1 region. Interestingly, predictions for less active compounds such as **1d**, **2d**, and **19d** (Figure 4B) orientated the structures upside down, with the oxadiazole-1-oxide in the S1′ region for compounds **1d** and **19d**, and the chlorophenyl substituent in the S3 region for compounds **2d** and **19d**. It is worth mentioning that the active compound **22d**, which is more “elongated” (aminophenyl-sulfonyl-aniline moiety bound to benzofuroxan) than the other analogues of the family, maintained the same hydrogen bonding interactions with the S3 region but through its amino group, while the oxadiazole-1-oxide occupies a new region near the active site, allowing a hydrogen bond interaction with THR25. Regarding the chalcone-related structures, the predicted binding mode for the active compounds in the series (**25d** and **27d**, Figure 4C) exhibited a similar orientation within the S3 region (forming hydrogen bonds with THR190), and the same non-polar interactions that were found for the active benzofuroxan derivatives. However, in this case, the hydroxyl and the naphthyl substituents are responsible for the interaction with the S3 and S1 regions, respectively. The less active compound **26d**, an isomer of **27d**, has a relative position of the carbonyl and hydroxyl groups that did not allow the same hydrogen bond interactions found for active structures and, as observed for inactive compounds of the benzofuroxan family, they inverted the orientation of the molecule into the active site (Figure 4D). The docking predictions for the active structure **28d** showed a different binding pose, perhaps due to its more compact shape (not shown). This isomer has the naphthyl group connected to the rest of the molecule by an alpha-carbon atom, as opposed to other active structures having a beta substitution. The compound was oriented near the catalytic dyad, forming hydrogen bonding interactions with CYS145 and other residues within the S1’ region. Finally, the binding pose suggested for the most active structure of the singletons set (**39d**, Table 11; Supplementary Figure S3) shared the position of the carbonyl group into the active site with the active compounds **25d** and **27d** but incorporated new lipophilic interactions with GLN189 and PRO168 residues through the chlorine substituents. Similarly, the phenanthridine group is located in the same region of the naphthyl moiety of compounds **25d** and **27d**, but in a different orientation, perhaps due to its larger size and ability to form a hydrogen bond interaction with GLU166, and a T-shaped *π*-stacking with HIS163.

## 4 Discussion

As in many other fields of science, artificial intelligence has contributed to speed-up and cheapening research and development processes in drug discovery. The access to large amounts of high-quality data for model training is, however, a persistent bottleneck in artificial intelligence-dependent approaches (Bittner and Farajnia, 2022). Bearing this in mind, and with the aim to contribute to the identification of small chemical compounds targeting the MPro from SARS-CoV-2, we embarked on a drug discovery campaign featuring strong computer and wet lab iteration.

Both our *in silico* and experimental validation results clearly show that the model ensemble used in the second prospective screening campaign possesses a much higher predictive value than the model ensemble applied in the first prospective screening. As the docking protocol did not vary between campaigns, the key to the different success rate in each *in silico* screening should be searched in the ligand-based models. There were five essential differences between the ligand-based models in the first and second campaigns, which are.

A) Increase in training set size, from 84 total compounds in the case of the first campaign to 160 compounds in the case of the second campaign, owing to the availability of data on new inhibitors at the time the second dataset was compiled.
B) Replacement of Dragon descriptors (first campaign) with Mordred descriptors (second campaign).
C) Use of more reliable data in the second campaign than in the first.
D) Use of iRaPCA to cluster and divide the dataset in the second campaign (instead of LibraryMCS plus k-means, which was used in the first campaign).
E) Use of LUDe to generate decoys (second campaign), instead of DUD-e (first campaign).

The use of Mordred descriptors instead of Dragon descriptors is unlikely to explain the different results across campaigns, because the pool of Dragon descriptors is larger and more diverse than those in Mordred. Similarly, while LUDe seems to generally perform slightly better than DUD-e in terms of the degree of embedding between the decoys and the queries, and also in terms of the doppelganger score, these differences are very small and unlikely to explain any substantial difference. iRaPCA provides excellent performance in the clustering of small molecules and may have provided a better representative sampling of the training data; this may be one of the reasons for the success of the second campaign. However, from our perspective, bearing in mind the well-known “garbage in, garbage out” principle (which, in essence, states that flawed, nonsense and/or mislabeled input data produce nonsense output), the most likely explanation of the good results in the second campaign (and the negative results in the first campaign) is the difference in the quality of the data used in one and the other. The second campaign was based entirely on data extracted from peer-reviewed articles or obtained in-house under highly standardized conditions. In contrast, little of the data used in the first screen (which was performed between July and October 2020, soon after the pandemic started) were extracted from published peer-reviewed articles. Note that an impressive volume of COVID-19-related literature was published in early 2020, encompassing a diversity of fields, from molecular biology to economics, from immunology to drug discovery. Possibly owing to the short timeframe, even published peer-reviewed papers contained substantial flaws or reached overly optimistic conclusions. A quick search in the Retraction Watch Database (http://retractiondatabase.org/) with a focus on retracted articles with the term ‘COVID-19″ in the title reveals that, so far, more than 50 items have been retracted. The reasons are diverse, but among the most frequent causes of retraction appear concerns about data, results, and methodological issues. Accordingly, we believe that the use of more reliable, less noisy data has resulted in models with (much) increased predictive power.

An additional and relevant outcome of our study was the identification of eleven compounds, many of them novel, with remarkable inhibitory activity against MPro. The hits displayed a broad chemical diversity that embraced a family of benzofuroxans (four hits), chalcones (three hits), benzoyl-thiazol (one hit), glycosides (one hit), flavonols (one hit) and one singleton, a (phenanthridinyl-methyl)-pyridazinone. Five out of the six MPro hits belonging to the first two families were also capable to impair SARS-CoV-2 replication *in vitro* at one or two digits μM concentrations.

Despite their broad biological activities and pharmacological interest (Chugunova and Burilov, 2017), so far there are no studies reporting the activity of benzofuroxans against SARS-CoV-2 and/or molecular targets thereof. Our study identified within this family four MPro inhibitors with nM IC_50_, two of them displaying low μM activity against SARS-CoV-2. Future research will address structural modifications at the heterocyclic ring with the aim of retaining or increasing anti-MPro/SARS-CoV-2 activity while improving the borderline selectivity and cancel out any potential genotoxicity associated with the benzofuroxan moiety (Cabrera et al., 2009).

The chalcone scaffold, present in several natural compounds, is characterized by having a highly reactive bond (i.e., *α*, *ß*-unsaturated ketone group) prone to undergo Michael’s addition, for instance, with nucleophylic cysteine residues. This scaffold attracted an early interest as source of potential inhibitors of SARS-CoV-2 proteases due to its well-known promiscuity to inhibit cysteine proteases and/or to block the replication of related coronaviruses (Raghav and Kaur, 2015; Park et al., 2016; Mathpal et al., 2022; Valipour, 2022). In fact, recent studies reported anti-MPro (Guterres Fernandes et al., 2023) and anti-SARS-CoV-2 activity (Duran et al., 2021) for different substituted chalcones containing homo- or hetero-nuclear aromatic rings. Except for the common chalcone scaffold, none of these molecules (mostly halogenated and/or furan-substituted) resembled the structure of the three potent hits (naphthalene-substituted) identified in our study. This suggests that the MPro active site can accommodate bulky groups attached to the chalcone structure, as further supported by our docking predictions. Though these results add value to the bio-potential of this scaffold, the marginal selectivity of our molecules is yet an issue to be addressed.

In plants, chalcones serve as primary substrates for the biosynthesis of flavonoids, which are polyphenolic compounds displaying a large diversity of biological functions and pharmacological activities (Wen et al., 2021). Several secondary metabolites or synthetic versions of these pythochemicals have been investigated for their anti-viral activity (for a thorough review see Badshah et al., 2021). A couple of these studies identified epigallocatechin gallate as inhibitor of MPro (Jang et al., 2020) and SARS-CoV-2 (Henss et al., 2021). Although not proposed as MPro hit by our *in silico* approach, screening of a small subset of flavonols extracted from grapes showed full inhibition of MPro activity by 25 μM epicatechin gallate (**5b**). The high similarity between epicatechin gallate and epigallocatechin gallate suggests that the first should be as active as the latter against SARS-CoV-2 (Henss et al., 2021). The quantitative contribution of MPro inhibition to the anti-SARS-CoV-2 activity of this type of flavonoids has not yet been studied and attaining target-selectivity may prove challenging for compounds with a remarkable polypharmacological reputability.

With respect to the glycosides family analysed, our data suggest that the conjugation of all OH groups from the sugar moiety with benzoyl, phenyl and/or acetyl groups is important for conferring inhibitory activity against MPro. Despite its low anti-MPro activity (IC_50_ 20 μM), the hit from this family (**7a**) displayed a minor antiviral activity and lacked cytotoxicity at the highest concentration tested (50 μM). For this compounds class, the optimization of MPro inhibition should be a priority that, if successful, may yield analogues with increased potency towards SARS-CoV-2.

On the other hand, the low IC_50_ against MPro of the (phenanthridinyl-methyl)-pyridazinone singleton (**39d**) is a good starting point for exploring new chemical spaces and functional groups that would enable a structure-activity relationship analysis.

Finally, is worth to mention that our study also revealed many compounds -from different chemical families- with anti-MPro activity borderline to the quantitative definition of hit. As in a fragment-based approach, this information may be useful to tests compounds’ combinations, and eventually, propose the synthesis of novel hybrid molecules with improved affinity for the molecular target.

## Data Availability

The original contributions presented in the study are included in the article/Supplementary Materials and further inquiries can be directed to the corresponding authors.
