# Supplementary material for: Garbage in, garbage out: how reliable training data improved a virtual screening approach against SARS-CoV-2 MPro
Source: Front Pharmacol. 2023 Jun 22;14:1193282. doi: 10.3389/fphar.2023.1193282 (PMC10323144; doi:10.3389/fphar.2023.1193282)
Supplement: Supplementary file 2 [file Table1.DOCX]

**Supplementary** **Figure S1**. Heatmap showing the molecular diversity of the dataset.


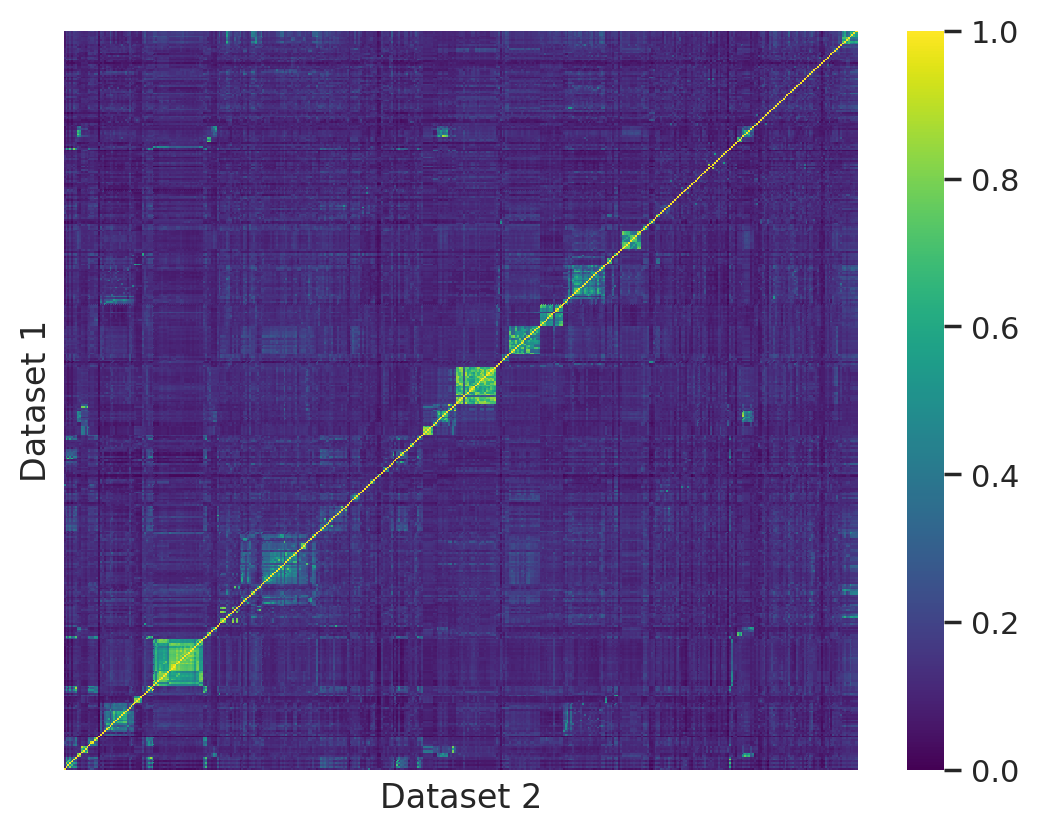


**Supplementary Figure S2.** Pose prediction accuracy, measured as the RMSD (Å), using AutoDock4-GPU for re-docking and cross-docking simulations across the whole set of structures and ligands.


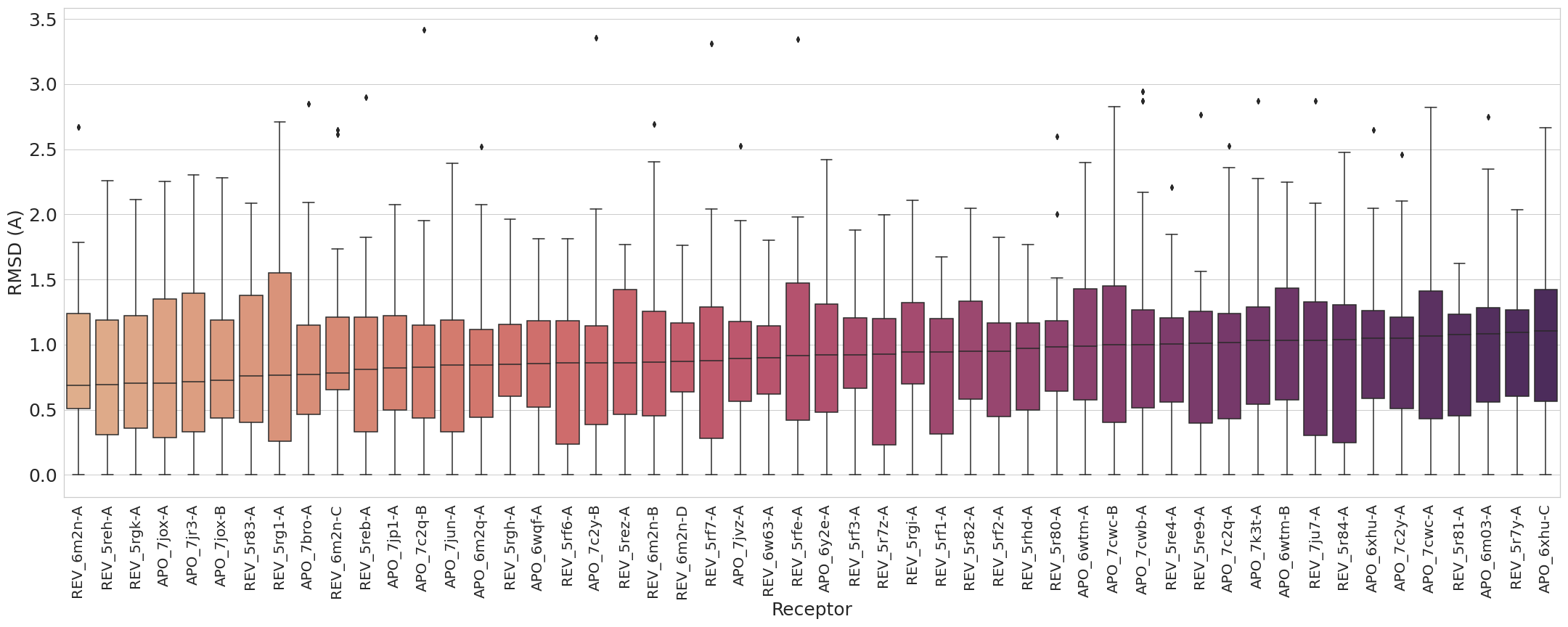


**Supplementary Figure S3.** Binding pose predicted by docking for compound **39d**, the most active structure of the singletons set.


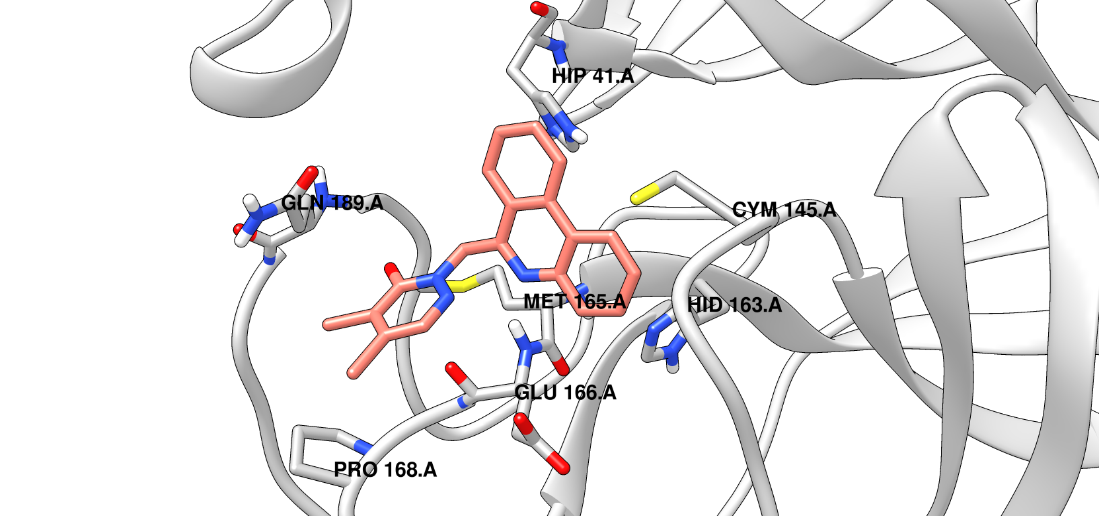


## Supplementary Table S1. Experimental screening (10 µM compound) against MPro from SARS-CoV-2 of hit candidates predicted by the first virtual screening (ligand model: light grey background; ligand + receptor model: dark grey background) and related analogues.

| **Category** | **Compound code** | **Structure** | **% activity MPro or *IC50* (μM)** | | |
| --- | --- | --- | --- | --- | --- |
|  |  |  | **10 μM** | **25 μM** | |
| BBHPP ^a^  BBHPP ^a^  BBHPP ^a^ | 9a |  | 94.6 ± 3.3 | | ND |
|  | 10a |  | 105.4 ± 6.1 | | ND |
|  | 11a | 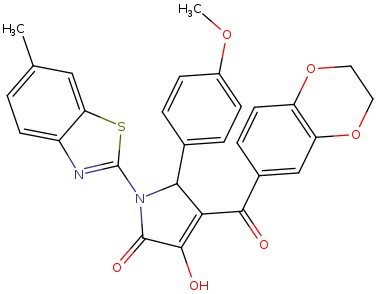 | 103.5 ± 1.1 | | ND |
|  | 12a | 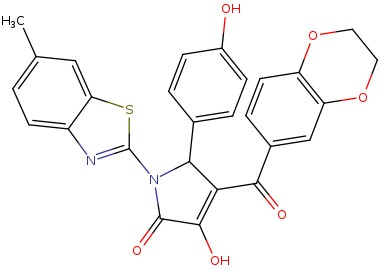 | 108.4 ± 2.1 | | ND |
|  | 13a | 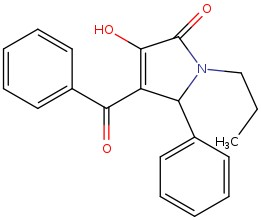 | 98.6 ± 0.9 | | ND |
|  | 14a |  | 105.4±0.5 | | ND |
|  | 15a | 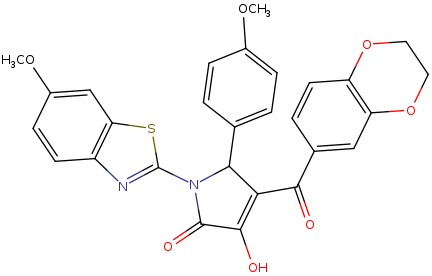 | 104.6 ± 2.2 | | ND |
|  | 16a | 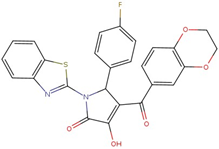 | 102.7 ± 5.2 | | ND |
|  | 17a | 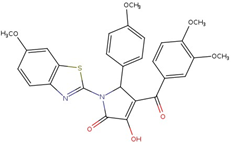 | 83.8 ± 1.8 | | 88.9 ± 8.4 |
|  | 18a | 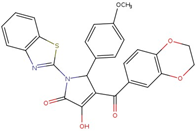 | 104.2 ± 4.7 | | ND |
|  | 19a |  | 98.1 ± 1.1 | | ND |
|  | 20a |  | 95.0 ± 5.2 | | ND |
|  | 21a | 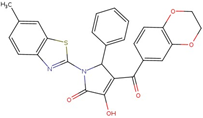 | 101.8 ± 0.8 | | ND |
|  | 22a | 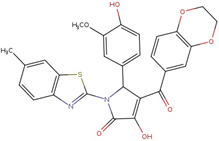 | 106.4 ± 1.8 | | ND |
|  | 23a | 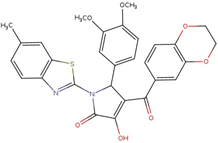 | 103.6 ± 6.1 | | ND |
|  | 24a | 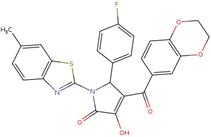 | 95.8 ± 1.7 | | ND |
|  | 25a | 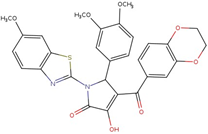 | 106.3 ± 1.0 | | ND |
|  | 26a | 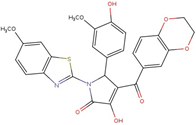 | 101.0 ± 1.7 | | ND |
|  | 27a | 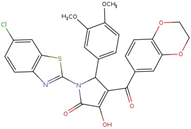 | 99.3 ± 7.9 | | ND |
|  | 28a | 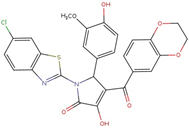 | 96.2 ± 1.9 | | ND |
| BAZ^b^  BAZ^b^ | 29a |  | 99.7 ± 5.1 | | ND |
|  | 30a |  | 118.0 ± 4.0 | | ND |
|  | 31a | 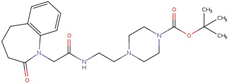 | 115.1 ± 6.6 | | ND |
|  | 32a | 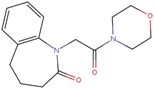 | 111.1 ± 2.3 | | ND |
|  | 33a | 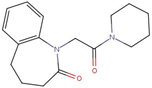 | 108.6 ± 3.4 | | ND |
|  | 34a | 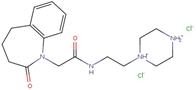 | 104.8 ± 3.7 | | ND |
|  | 35a | 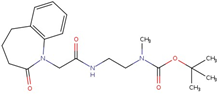 | 114.2 ± 3.8 | | ND |
|  | 36a | 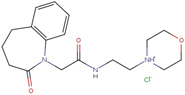 | 110.5 ± 3.8 | | ND |
|  | 37a | 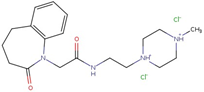 | 103.8 ± 2.1 | | ND |
|  | 38a | 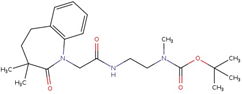 | 110.4 ± 3.1 | | ND |
|  | 39a | 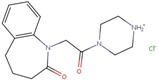 | 110.7 ± 3.5 | | ND |
|  | 40a | 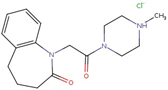 | 105.7 ± 1.4 | | ND |
|  | 41a | 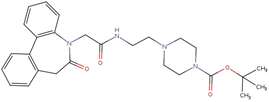 | 115.6 ± 3.4 | | ND |
|  | 42a | 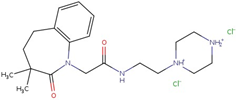 | 108.6 ± 4.0 | | ND |
|  | 43a | 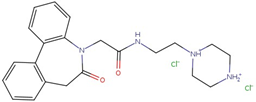 | 104.2 ± 1.6 | | ND |
|  | 44a | 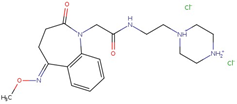 | 100.6 ± 3.8 | | ND |
| CP^c^  CP^c^ | 45a | 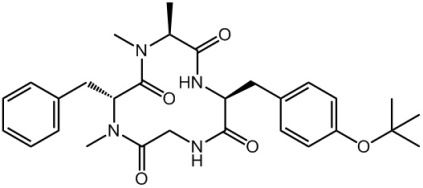 | 116.7 ± 1.2 | | ND |
|  | 46a | 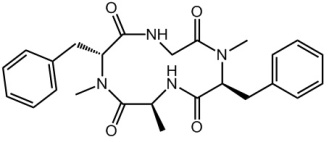 | 115.1 ± 6.6 | | ND |
|  | 47a | 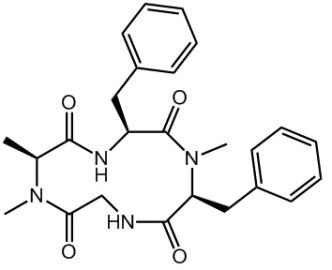 | 126.3 ± 6.0 | | ND |
|  | 48a * | 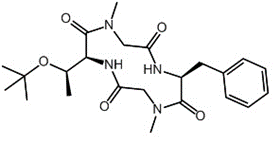 | 102.8 ± 1.6 | | 121.4 ± 2.9 |
|  | 49a |  | 104.4 ± 1.7 | | ND |
|  | 50a | 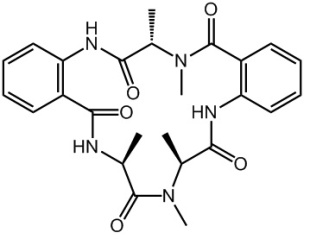 | 105.8 ± 6.7 | | 104.8 ± 7.3 |
|  | 51* | 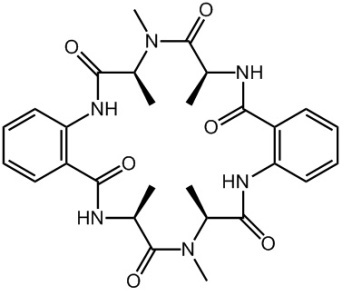 | ND | | 107.4 ± 5.7 |
|  | 52a * | 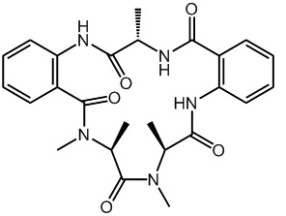 | ND | | 94.9 ± 2.4 |
| PM^d^  PM^d^  PM^d^  PM^d^ | 53a | 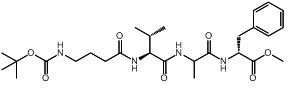 | 116.5 ± 2.9 | | 106.8 ± 0.1 |
|  | 54a | 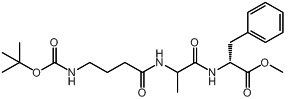 | 117.0 ± 1.0 | | 76.7 ± 1.6 |
|  | 55a |  | 100.0 ± 4.9 | | 83.2 ± 3.4 |
|  | 56a * |  | 99.8 ± 5.2 | | ND |
|  | 57a * |  | 107.9 ± 3.5 | | ND |
|  | 58a * |  | 101.2 ± 4.6 | | ND |
|  | 59a * | 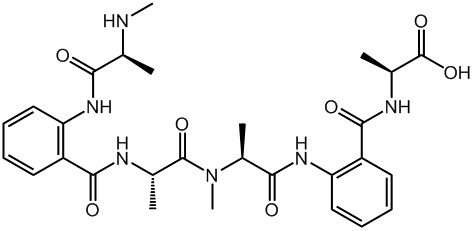 | ND | | 101.4 ± 4.8 |
|  | 60a * | 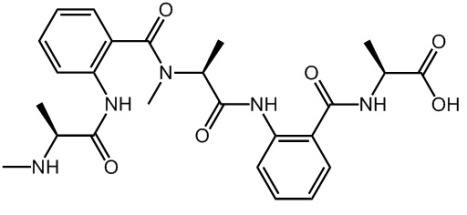 | ND | | 118.0 ± 3.9 |
|  | 61a * | 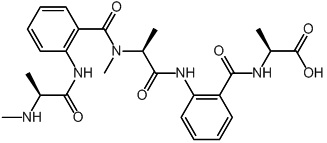 | ND | | 109.4 ± 6.7 |
|  | 62a * | 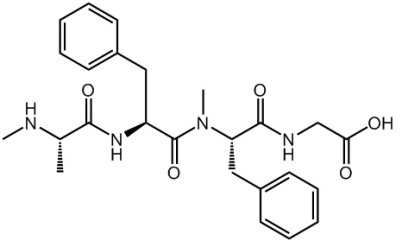 | ND | | 108.0 ± 1.5 |
|  | 63a * |  | ND | | 110.8 ± 0.4 |
|  | 64a * | 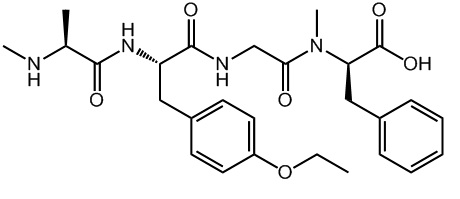 | ND | | 83.0 ± 30.3 |
|  | 65a * | 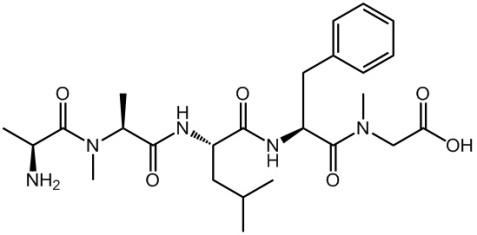 | ND | | 102,0±8,7 |
|  | 66a * | 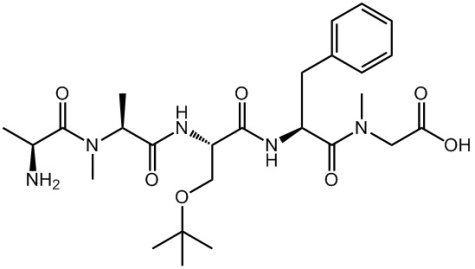 | ND | | 109.2 ± 9.8 |
|  | 67a * | 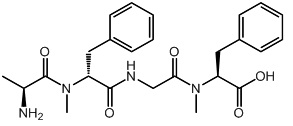 | ND | | 69.6 ± 0.9 |
|  | 68a * | 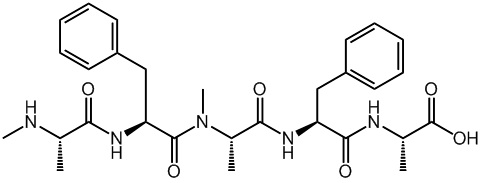 | ND | | 111.8 ± 3.6 |
|  | 69a * |  | ND | | interference |
|  | 70a * | 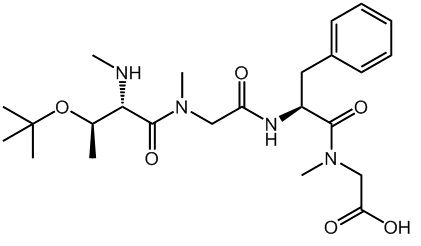 | ND | | 110.3 ± 0.2 |
|  | 71a * | 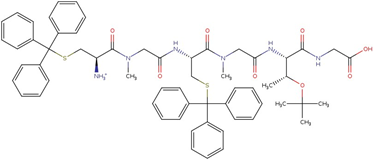 | ND | | 107.2 ± 1.2 |
|  | 72a * | 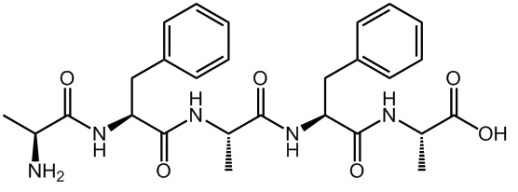 | ND | | 101.4 ± 4.8 |
|  | 73a * | 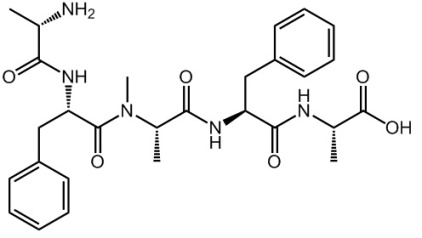 | ND | | 113.8 ± 7.9 |
|  | 74a * | 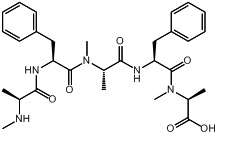 | ND | | 103.6 ± 5.8 |
|  | 75a * | 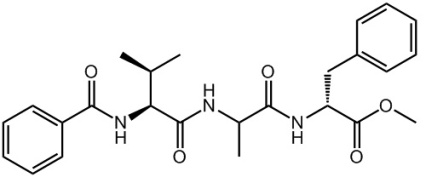 | ND | | 98.1 ± 2.5 |
|  | 76a | 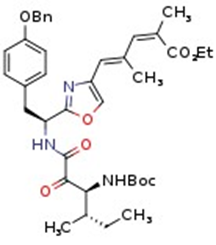 | ND | | 94.6 ± 4.7 |
|  | 77a | 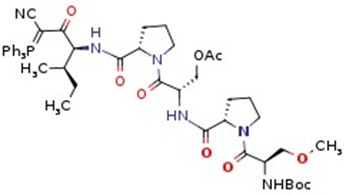 | ND | | 108.2 ± 2.4 |
| CH^e^  CH^e^ | 78a | 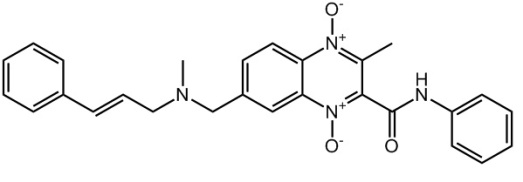 | 116.8 ± 1.1 | | 81.1 ± 3.3 |
|  | 79a | 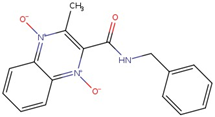 | 102.9 ± 1.4 | | 59.4 ± 3.6 |
| APPDA^f^  APPDA^f^ | 80a * |  | ND | | 100.0 ± 2.5 |
|  | 81a |  | 111.6 ± 2.3 | | ND |
|  | 82a |  | 107.7 ± 1.8 | | ND |
|  | 83a | 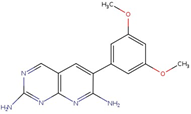 | 97.7 ± 1.6 | | ND |
|  | 84a | 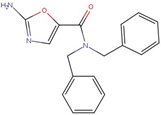 | 108.4 ± 2.2 | | ND |
|  | 85a | 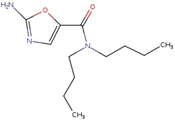 | 109.8 ± 2.8 | | ND |
|  | 86a | 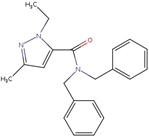 | 111.0 ± 2.4 | | ND |
|  | 87a | 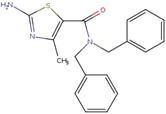 | 103.3 ± 1.0 | | ND |
|  | 88a * | 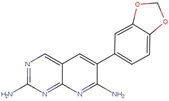 | 89.0 ± 2.3 | | 90.6 ± 1.4 |
|  | 89a * | 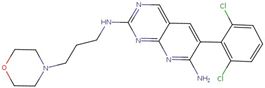 | 87.9 ± 5.7 | | interference |
|  | 90a * | 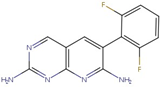 | 84.7 ± 3.2 | | interference |
|  | 91a |  | 120.1 ± 11.3 | | ND |
| ABTPC^g^  ABTPC^g^ | 92a |  | 114.6 ± 2.2 | | ND |
|  | 93a |  | 108.0 ± 0.9 | | ND |
|  | 94a |  | 99.1 ± 4.0 | | ND |
|  | 95a |  | 105.1 ± 5.7 | | ND |
|  | 96a |  | 110.7 ± 4.1 | | ND |
|  | 97a |  | 100.1 ± 2.9 | | ND |
|  | 98a |  | 114.4 ± 2.0 | | ND |
|  | 99a |  | 110.4 ± 3.9 | | ND |
|  | 100a |  | 104.3 ± 1.3 | | ND |
|  | 101a |  | 103.3 ± 1.0 | | ND |
|  | 102a |  | 92.8 ± 9.6 | | ND |
|  | 103a |  | 97.2 ± 9.6 | | ND |
| Pyrimidin-based | 104a | 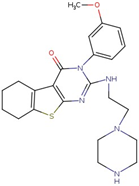 | 104.7 ± 1.4 | | ND |
| AA^h^  AA^h^ | 105a * | 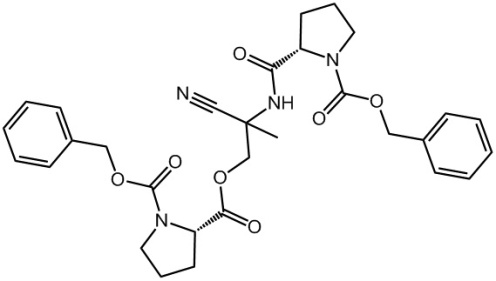 | ND | | 108.2 ± 3.1 |
|  | 106a * | 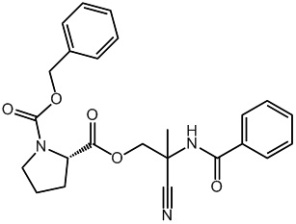 | 102.6 ± 8.4 | | 89,7±2,3 |
|  | 107a * | 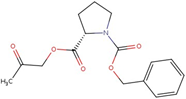 | ND | | 101.7 ± 1.5 |
|  | 108a * | 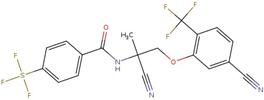 | ND | | 103.6 ± 5.1 |
|  | 109a * | 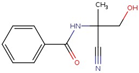 | ND | | 105.4 ± 2.4 |
| VL^i^ | 110a * | 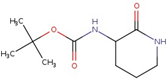 | ND | | 108.8 ± 2.2 |
|  | 111a * | 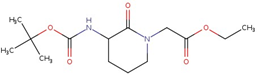 | ND | | 109.2 ± 4.4 |
|  | 112a * | 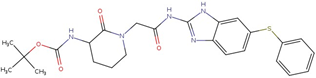 | ND | | 109.3 ± 3.3 |
| Corey lactone derivative | 113a * | 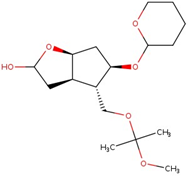 | ND | | 111.8 ± 11.2 |
| monocyclic β-lactam  monocyclic β-lactam | 114a * |  | 84.6 ± 0.4 | | 94.2 ± 2.0 |
|  | 115a * |  | 92.8 ± 2.4 | | 94.9 ± 0.3 |
|  | 116a * |  | 94.2 ± 3.4 | | 86.9 ± 1.0 |
|  | 117a * |  | 86.9 ± 2.9 | | ND |
|  | 118a * |  | 90.9 ± 1.4 | | ND |
| Glycosides  Glycosides  Glycosides  Glycoside | 119a * |  | NA | | NA |
|  | 120a * |  | NA | | NA |
|  | 121a * |  | NA | | NA |
|  | 122a * |  | ND | | 109.6 ± 1.1 |
|  | 123a * |  | ND | | 112.7 ± 1.3 |
|  | 124a * |  | ND | | 107.8 ± 5.0 |
|  | 125a * |  | ND | | 118.2 ± 3.7 |
|  | 126a * |  | ND | | 103.9 ± 0.7 |
|  | 127a * |  | ND | | 124.7 ± 10.9 |
|  | 128a * |  | ND | | 109.4 ± 1.4 |
|  | 129a * |  | ND | | 113.1 ± 4.9 |
|  | 130a * |  | ND | | 118.5 ± 5.7 |
|  | 131a * |  | ND | | 112.8 ± 8.8 |
|  | 132a * |  | ND | | 107.7 ± 5.1 |
|  | 133a * |  | ND | | 111.5 ± 5.0 |
|  | 134a * |  | ND | | 105.5 ± 4.8 |
|  | 135a * |  | ND | | 109.5 ± 3.9 |
|  | 136a * |  | ND | | 107.1 ± 2.9 |
|  | 137a * |  | ND | | 60.9 ± 1.5 |
|  | 138a * |  | ND | | 106.7 ± 1.5 |
|  | 139a * |  | ND | | 67.0 ± 2.5 |
|  | 140a * |  | ND | | 74.7 ± 3.9 |
|  | 141a * |  | ND | | 76.3 ± 2.2 |
|  | 142a * |  | ND | | 60.2 ± 2.1 |
| 2-amine-ε-caprolactam | 143a * | 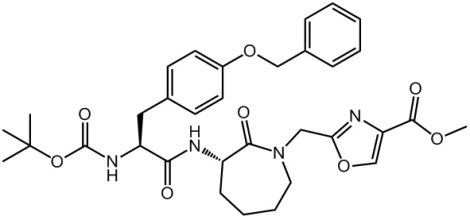 | ND | | 112.2 ± 4.6 |
|  | 144a * | 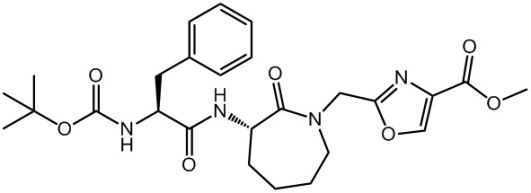 | ND | | 98.8 ± 1.4 |
| Tocopherol-mimetic bisamide (singleton) | 145a * | 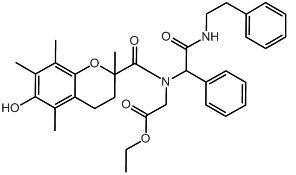 | ND | | 87.4 ± 19.9 |
| SFH^j^ | 146a * | 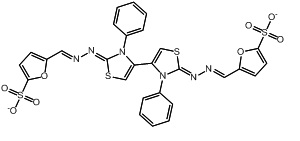 | ND | | 65.6 ± 10.9 |

## ^a^ BBHPP: 1-(benzo[*d*]thiazol-2-yl)-4-benzoyl-3-hydroxy-5-phenyl-1*H*-pyrrol-2(5*H*)-one

^b^ BAZ: benzazepinone

^c^ CP: cyclopeptides

^d^ PM: peptidomimetics

^e^ CH: chalcones

^f^ 6-arylpyrido[2,3-*d*]pyrimidine-2,7-diamine derivatives

^g^ ABTPC: N-(2-aminobenzo[d]thiazol-6-yl)-1H-pyrrole-2-carboxamide

^h^ AA: amino-acetonitrile derivatives

^i^ VL: Valerolactam derivatives (with 111a being a Valerolactam-fenbendazole hybrid)

^j^ SFH: sulfonatofuran hydrazine bithiazole

## * data not used to train the model MIN-22

ND: not determined

NA: not available for testing

## Supplementary Table S2. Experimental screening against MPro from SARS-CoV-2 not predicted as hit candidates by virtual screening but selected randomly or empirically, and not used to train the search models. Hits (MPro inhibition ≥50%) are labelled in grey background.

| **Category** | **Compound code** | **Structure** | **% activity MPro at 10 μM or *25 μM*** |
| --- | --- | --- | --- |
|  |  |  |  |
| Flavonols | 1b | Non-purified extract | *94.8 ± 5.3* |
|  | 2b | Non-purified extract | *98.0 ± 6.3* |
|  | 3b | Non-purified extract | *92.1 ± 2.8* |
|  | 4b |  | *88.7 ± 0.6* |
| Pyrazoles  Ppyrazoles  Pyrazoles  Pyrazoles  Pyrazoles | 1c |  | interference |
|  | 2c |  | interference |
|  | 3c |  | 107.5 ± 34.4 |
|  | 4c |  | 130.0 ± 6.7 |
|  | 5c |  | 156.6 ± 3.9 |
|  | 6c |  | 82.9 ± 1.0 |
|  | 7c |  | 90.4 ± 0.9 |
|  | 8c |  | 90.9 ± 2.2 |
|  | 9c |  | 80.4 ± 0.5 |
|  | 10c |  | 118.7 ± 8.4 |
|  | 11c |  | 102.3 ± 3.3 |
|  | 12c | 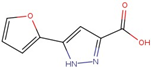 | 86.0 ± 8.0 |
|  | 13c |  | 94.4 ± 2.3 |
|  | 14c |  | 69.4 ± 3.6 |
|  | 15c |  | 101.6 ± 4.0 |
|  | 16c |  | 100.1 ± 0.5 |
|  | 17c |  | 94.5 ± 0.7 |
|  | 18c |  | 94.4 ± 0.4 |
|  | 19c |  | 85.8 ± 1.0 |
|  | 20c |  | 83.0 ± 2.9 |
|  | 21c |  | 84.6 ± 3.5 |
|  | 22c |  | 88.9 ± 6.4 |
|  | 23c |  | 71.8 ± 1.9 |
|  | 24c |  | 88.2 ± 3.1 |
|  | 25c |  | 104.9 ± 0.0 |
|  | 26c |  | 166.7 ± 9.5 |
|  | 27c |  | 172.1 ± 12.5 |
|  | 28c |  | 175.8 ± 11.0 |
|  | 29c |  | *83.1 ± 9.4* |
|  | 30c |  | *86.0 ± 8.7* |
|  | 31c |  | *88.3 ± 15.9* |
|  | 32c |  | *91.9 ± 9.6* |
|  | 33c |  | *91.1 ± 8.9* |
|  | 34c |  | *88.0 ± 11.0* |
|  | 35c |  | *85.5 ± 5.5* |

**Supplementary information of the best five individual models in each campaign**

**In silico first screening campaign**

**MODEL 324**

**DF** = 2.02804 + 0.61388***NsCl** - 2.85484***BIC1** - 0.47735***F08[N-Cl]** + 0.16156***CATS2D_03_DD** + 0.03877***CATS2D_06_AL** - 0.01329***ATSC3m** + 0.06373***nCconj**

where **NsCl** is the number of atoms of type sCl, **BIC1** is the Bond Information Content index (neighborhood symmetry of 1-order), **F08[N-Cl]** is the Frequency of N - Cl at topological distance 8, **CATS2D_03_DD** correspond to the CATS2D Donor-Donor at lag 03, **CATS2D_06_AL** correspond to the CATS2D Acceptor-Lipophilic at lag 06, **ATSC3m** refers to the Centred Broto-Moreau autocorrelation of lag 3 weighted by mass and **nCconj** is the number of non-aromatic conjugated C(sp2).

**MODEL 644**

**DF** = -0.637545 + 0.009443***P_VSA_m_4** + 0.046745***CATS2D_05_AL** + 0.193794***CATS2D_03_DD** - 0.382247***B04[C-S]** + 0.163686* **NsssN** + 0.489085***GATS6**

where **P_VSA_m_4** refers to P_VSA-like on mass (bin 4), **CATS2D_05_AL** is the CATS2D Acceptor-Lipophilic at lag 05, **CATS2D_03_DD** is the CATS2D Donor-Donor at lag 03, **B04[C-S]** correspond to the Presence/absence of C - S at topological distance 4, **NsssN** is the Number of atoms of type sssN and **GATS6m** is the Geary autocorrelation of lag 6 weighted by mass.

**MODEL 739**

**DF** = -1.1875 + 0.7715*F03**[O-Cl]** + 0.3338***Eig05_AEA(bo)** + 0.2337***NdsCH** + 0.3371***GATS7e**

where **F03[O-Cl]** is the Frequency of O - Cl at topological distance 3, **Eig05_AEA(bo)** refers to the eigenvalue n. 5 from augmented edge adjacency matrix weighted by bond order, **NdsCH** is the Number of atoms of type dsCH and **GATS7e** is the Geary autocorrelation of lag 7 weighted by Sanderson electronegativity.

**MODEL 510**

**DF** = 0.4441 + 0.7622***Cl-086** + 0.2130***SM08_AEA(dm)** + 0.5578***nROCON** - 0.2147***B04[C-S]**

where **Cl-086** refers to Cl atom attached to C1(sp3), **SM08_AEA(dm)** is the spectral moment of order 8 from augmented edge adjacency mat. weighted by dipole moment, **nROCON** correspond to the number of (thio-) carbamates (aliphatic) and **B04[C-S]** is the Presence/absence of C - S at topological distance 4.

**MODEL 390**

DF = -10.09896 + 0.72045***nCH2RX** - 5.93178***SpMaxA_B(m)** - 0.81397***MATS7m** - 0.61420***IC1** + 13.40149***Me** + 0.21545***B08[N-N]** - 0.04176***nCrs** - 0.17032***NsF**

where **nCH2RX** is the number of CH2RX, **SpMaxA_B(m)** correspond to the normalized leading eigenvalue from Burden matrix weighted by mass, **MATS7m** is the Moran autocorrelation of lag 7 weighted by mass, **IC1** refers to the Information Content index (neighborhood symmetry of 1-order), **Me** is the mean atomic Sanderson electronegativity (scaled on Carbon atom), **B08[N-N]** is the Presence/absence of N - N at topological distance 8, **nCrs** is the number of ring secondary C(sp3) and **NsF** indicates the Number of atoms of type sF.

**In silico second screening campaign**

**MODEL 25**

DF = -1.6239 + 0.2421***NsCl** - 0.0003***ATSC2m** + 0.3449***NaaS** - 0.0226***VSA_EState4** + 0.1852***BCUTi-1l** + 0.4483***SddsN** - 0.0494***nARing** + 0.1496***NdO** - 0.1101***NdssC** - 0.2233***nAcid**

where **NsCl** is the number of sCl (*s* for single bond), **ATSC2m** is the averaged and centered Moreau-Broto autocorrelation of lag 2 weighted by atomic mass, **NaaS** is the number of sulfur atoms linked to two atoms that belong to an aromatic ring, **VSA_EState4** is the VSA Estate Descriptor 4, **BCUTi-1l** is the first lowest eigenvalue of Burden matrix weighted by ionization potential, **SddsN** is the sum of -N<< (where – denotes a single bond and << denotes either two double bonds or two resonant single/double bonds, as in a nitro group), **nARing** symbolizes the aromatic ring count, **NdO** is the number of =O (with = representing a double bond), **NdssC** is the number of =C< (= denotes a doble bond, and < denotes two single bonds) and **nAcid** is the acidic group count

**MODEL 361**

DF = 6.0528 + 0.1968* **nCl** - 0.0021* **ATSC2Z** - 0.1729* **NsNH2** + 0.1334* **SaasN** - 0.0331***ATSC3p** + - 0.00003* **ATS0m** + 0.0067* **SdO** - 0.0340* **AATS4i** - 0.2436* **nFARing** - 0.0031* **SMR_VSA7**

where **nCl** is the number of Cl atoms, **ATSC2Z** is the centered Moreau-Broto autocorrelation of lag 2 weighted by atomic number, **NsNH2** is the number of sNH2 (s symbolizes a single bond), SaasN is the sum of aasN (a represents an atom that belongs to an aromatic ring, s represents a single bond, N denotes a nitrogen atom), **ATSC3p** is the centered Moreau-Broto autocorrelation of lag 3 weighted by atomic polarizability, **ATS0m** symbolizes the Moreau-Broto autocorrelation of lag 0 weighted by atomic mass, **SdO** is the sum of dO (d represents a double bond, O represents an oxygen atom), **AATS4i** is the averaged Moreau-Broto autocorrelation of lag 4 weighted by atomic ionization potential, **nFARing** is the aromatic fused ring count, and **SMR_VSA7** is the MOE MR VSA Descriptor 7.

**MODEL 77**

DF = 0.5121 + 0.5421* **nCl** - 0.0276***AATSC2m** - 0.0292* **EState_VSA9** + 0.1539* **NaasN** + 0.0315* **SssO** - 0.2552* **nAcid** - 0.0003***TMPC10** + 0.0122* **SlogP_VSA8** + 0.0594* **NdO**

where **nCl** is the number of Cl atoms, **AATSC2m** is the averaged and centered Moreau-Broto autocorrelation of lag 2 weighted by atomic mass, **EState_VSA9** is the EState VSA Descriptor 9, **NaasN** is the number of aasN (a represents an atom that is member of a aromatic ring, s represents a single bond, N denotes a nitrogen atom), **SssO** is the sum of ssO (s being a single bond and O representing an oxygen atom), **nAcid** is the acidic group count, **TMPC10** is the total path count of order 10^th^, **SlogP_VSA8** is the MOE logP VSA Descriptor 8, and **NdO** is the number of =O (= denotes a double bond).

**MODEL 273**

DF = 1.1634 + 0.9300* **nCl** - 0.5821* **IC1** + 0.0001* **ATSC5v** - 0.0363* **EState_VSA9** + 0.0332* **SssO** -0.0001* **ATSC0m** - 0.2570* **nFARing** - 0.0858* **SlogP_VSA7** + 0.0345* **MIC2** - 0.0316* **ATSC2p** + 0.0202***MID_N**

where **nCl** denotes the number of Cl atoms, **IC1** symbolizes the neighborhood information content of first order, **ATSC5** is the centered Moreau-Broto autocorrelation of lag 5 weighted by van der Waals volume, **EState_VSA9** is the EState VSA Descriptor 9, **SssO** is the sum of ssO (s representing a single bond and O representing an oxygen atom), **ATSC0m** is the centered Moreau-Broto autocorrelation of lag 0 weighted by atomic mass, **nFARing** is the aromatic fused ring count, **SlogP_VSA7** is the MOE logP VSA Descriptor 7, **MIC2** is the modified information content of second order, **ATSC2p** is the centered Moreau-Broto autocorrelation of lag 2 weighted by atomic polarizability, and **MID_N** is the molecular ID on N atoms.

**MODEL 442**

DF = 1.8942 - 0.1024* **AATSC2Z** - 0.0601***nHBDon** + 0.1600***ETA_beta_ns_d** - 0.0347* **SaaN** + 0.1062* **SaasN** + 0.2747* **n6aHRing** + 0.0532* **C1SP3** + 0.4222* **n9FaRing** - 0.1196* **Xp-6d** - 0.2421* **Lipinski** + 0.0002* **ATSC7v** + 0.0091* **SdO** + 0.1179* **ATSC3pe** - 0.0036* **ATSC3dv** - 0.5119* **GATS4Z** - 0.2242***IC1**

where **ATSC2Z** is the averaged and centered Moreau-Broto autocorrelation of lag 2 weighted by atomic number, **nHBDon** is the number of hydrogen bond donors, **ETA_beta_ns_d** is the delta contribution to valence electron mobile count, **SaaN** is the sum of aaN (a being an atom of an aromatic system, N being a nitrogen atom), **SaasN** is the sum of aasN (a, again, representing an atom that participates in an aromatic ring, s stands for a single bond), **n6aHRing** is the of 6-membered aromatic hetero ring count, **C1SP3** is a SP3 carbon bound to other carbon, **n9FaRing** is the 9-membered aromatic fused ring count, **Xp-6d** is the Chi path of 6^th^ order weighted by sigma electrons, **Lipinski** is a binary descriptor that indicates if the compound fall within the Lipinski rule of five (1) or not (0), **ATSC7v** is the centered Moreau-Broto autocorrelation of lag 7 weighted by van der Waals volume, **SdO** is the sum of dO (d being a double bond, O representing an oxygen atom), **ATSC3pe** is the centered Moreau-Broto autocorrelation of lag 3 weighted by Pauling electronegativity, **ATSC3dv** is the centered Moreau-Broto autocorrelation of lag 3 weighted by the number of valence electrons, **GATS4Z** is the Geary coefficient of lag 4 weighted by atomic number, and **IC1** is the neighborhood information content of 1^st^ order.
